# Nutritional, Ethical and Ecological Aspects of Cultured Meat with Particular Emphasis on Functional Food Production: A Comprehensive Literature Review

**DOI:** 10.3390/foods15050891

**Published:** 2026-03-05

**Authors:** Marian Gil, Mariusz Rudy, Paulina Duma-Kocan, Renata Stanisławczyk, Dariusz Dziki

**Affiliations:** 1 Department of Agricultural Processing and Commodity Science, Institute of Food Technology and Nutrition, Faculty of Technology and Life Sciences, University of Rzeszów, Zelwerowicza 4, 35-601 Rzeszow, Poland; mgil@ur.edu.pl (M.G.); pduma@ur.edu.pl (P.D.-K.); rstanislawczyk@ur.edu.pl (R.S.); 2 Department of Thermal Technology, University of Life Sciences in Lublin, Głęboka 31, 20-612 Lublin, Poland

**Keywords:** cultured meat, cultured meat scaffolds, meat production, meat quality, farmed meat

## Abstract

This manuscript was developed to present a comprehensive analysis of the solutions and conditions used in the production of cultured meat (CM). This study addressed the following research question: What are the reasons for the development of CM? The aim of the study was to conduct a thorough review of the scientific literature on issues related to the production and quality of CM, as well as methods used to improve it, and to systematize these issues. Issues related to shaping the chemical composition and nutritional value towards functional foods, as well as the organoleptic properties and safety of CM, were presented. Issues related to consumer acceptance of CM were discussed. Further issues concerned CM as a market product, including the advantages and barriers associated with acquiring and maintaining markets. Key development conditions were discussed, such as the need to improve the quality of CM, technological development, and lower production costs. Socioeconomic challenges, such as the risk of deepening economic inequalities between countries and social classes, and the potential consequences for farmers, consumers, and rural populations, were also considered.

## 1. Introduction

Meat, usually described as the flesh of an animal meant for human consumption, incorporates a wide range of edible components, including lean muscle tissue and adipose tissue [1]. Meat is an important part of the human diet, associated with more than just culinary pleasure and fullness. Beyond its nutritional relevance, meat has a symbolic function in human culture, culinary traditions, and social events. It is associated with social distinction and celebration [2,3]. The increasing demand for meat protein, combined with the restrictions on resources such as land, water, and energy required for conventional animal production, highlights the critical need to discover more sustainable alternatives. Because animal agriculture contributes to worldwide environmental degradation, one strategy is to limit meat consumption [4] by substituting it with meat substitutes [5]. Furthermore, there is a growing disparity between future demand for protein sources, and the current capacity to supply meat [6]. To bridge this gap, alternative meat products—developed through cellular agriculture and plant-based technologies—have emerged as promising solutions [7].

Cultured meat (CM) can be defined as animal protein produced by in vitro animal cell culture techniques involving the steps of animal cell isolation, proliferation or expansion, cell differentiation in a bioreactor with a nutrient-rich medium into tissues that resemble traditional meat in both structure and function [8,9,10] and then processed in an aseptic laboratory or factory environment [11,12,13,14]. The main goal of producing laboratory-grown meat is to reconstruct the complex muscle structure of an animal using a small number of cells [15]. Laboratory-grown meat, also known as clean meat, synthetic meat, laboratory-grown meat, cell-cultured meat, or in vitro meat [16]. Muscle fibers cultured in vitro may differ in their degree of maturity compared to muscles from living domestic animals [17]. Consequently, in vitro CM may exhibit different sensory and processing properties compared to conventionally produced meat [18,19].

CM can be produced to produce full-sized meat products (e.g., steaks and whole cuts) or can be used to create processed meats such as hamburger patties or cooked sausage [16]. In 2013, the first prototype of a hamburger-shaped CM was presented. The hamburger was based on 10,000 strips containing muscle tubes designed in a hydrogel. However, the modified muscle-like tissues also required the addition of colorants (beetroot juice), flavorings (saffron and caramel), and texturizers (breadcrumbs and binder) to make the patty resemble a hamburger [18]. Currently, many companies are working on producing CM and selling it in the near future. The method of producing CM differs significantly from conventional animal farming and is supported by some political, scientific, and religious circles [20].

The aim of the study was to provide a comprehensive review of the scientific literature presenting issues related to the production and quality of CM, as well as solutions used to improve it, and to systematize them. Issues related to shaping: the chemical composition and nutritional value towards functional foods, as well as the organoleptic properties and safety of CM, were presented.

## 2. Materials and Methods

This work was the result of prior reading and detailed analysis of scientific publications on the issue of CM as an alternative to traditional meat in the face of growing demand, the environmental impact of intensive slaughter animal production methods, and ethical concerns related to breeding conditions and slaughter itself. The authors explore the determinants of CM development and production. Particular attention is paid to CM quality, given its critical importance for the development of meat production and consumer acceptance. To this end, attention is paid to shaping the appropriate structure of CM through the use of natural raw materials. This allows for the optimization of quality and, moreover, the enrichment and modification of CM’s properties as a functional food product.

**Figure 1:**
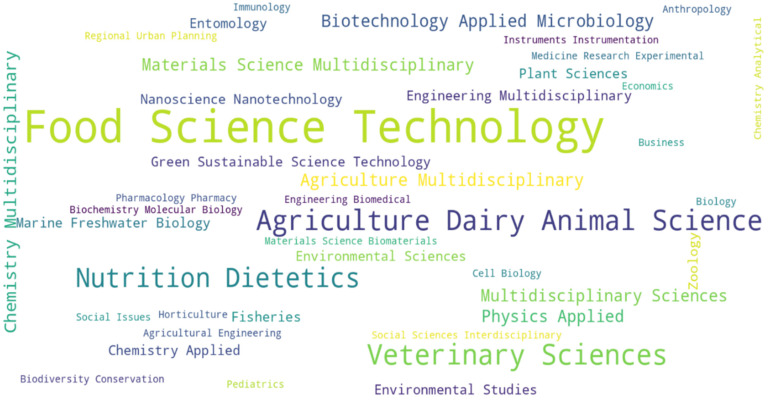
Thematic areas of journals publishing papers on CM.

The analysis of the scientific literature for this article took place from January to September 2025. The Scopus and Web of Science databases were used for this purpose. The search engines used the keyword sequences “cultured meat + nutritional aspects,” “cultured meat + ecological aspects,” and “cultured meat + nutritional ethical aspects.” The researchers analyzed the search results based on the topics covered and the study objectives. Scientific papers on CM represented the thematic areas presented in Figure 1. The initial search period covered the years 2020–2026; duplicates were removed from the imported results. The next step was to review titles and abstracts for relevance to the paper’s topic and exclude unrelated papers. The full-text assessment of publication eligibility took into account methodology and comparability with other works. Previous publications were included for a more comprehensive analysis of the issues discussed. From the analyzed group of publications, 440 were selected, considered to be the most relevant to the topic and purpose of the study.

The selection of articles for review was based on three criteria. The first group comprised studies related to the development of CM production technologies, taking into account quality engineering issues, such as the 3D structure of CM and the types of raw materials used to shape it. The second group consisted of publications focusing on CM quality research, including chemical composition, nutritional value, texture, and sensory properties. The next group of issues concerned CM as a market product: consumer preferences, advantages and barriers, and product safety. The Zotero 7.0.27 bibliography manager was used to collect, organize, and cite scientific sources

## 3. Determinants of CM Production

While overall meat consumption continues to increase, particularly due to rising incomes in developing countries, there is a significant shift in consumer attitudes, especially in developed countries [21]. In countries like the United States and throughout Europe, there is an increasing trend toward reduced meat consumption, particularly among younger groups [22].

Between 1998 and 2018, global meat consumption, driven primarily by population growth, increased by 58%, from about 200 million tonnes to 360 million tonnes. Income growth had the largest impact on per capita meat consumption. Meat consumption in China increased by 72% between 1998 and 2018, accounting for 34% of the global increase in meat consumption, which ranged from about 50 kg to 79 kg per person [23]. A similar trend occurred in Indonesia, where consumption increased from around 19 kg to 39 kg/person. Strong economic and demographic growth in Indonesia contributed to this increase. Furthermore, meat consumption in Australia increased from about 99 to 109 kg per capita and increased from about 110 to 119 kg per capita in the United States. Furthermore, many countries in Africa are currently experiencing population growth [24]. In recent decades, the expansion of the agricultural sector has resulted in half of the world’s habitable land being devoted to agriculture, three-quarters of which is used for growing cattle or crops for animal feed [25].

Aquaculture is currently the fastest-growing food production technology in terms of volume, with forecasts that it will double production by 2050 [26]. In low- and middle-income Asian and African countries, fish and seafood account for more than 20% of animal protein [27]. Fish fillets, an integral component of these food systems, make a significant contribution to income and nutrition, particularly in underdeveloped countries. Despite the fact that fish play a vital part in global nutrition, supplying rising demand is becoming increasingly difficult. Overfishing has resulted in 33.1% of fish stocks being exploited beyond sustainable yields, causing certain populations to decline [28,29]. This needs the development of alternate seafood sources to accommodate rising demand while lowering pressure on wild stocks and traditional aquaculture systems [30].

Economic development and urbanization have affected middle-class dietary patterns toward more opulent meals as part of the globalization process. This is understandable given that meat is a nutrient-dense, nutritious diet that contains all nine essential amino acids, six conditionally essential amino acids, and vital minerals and vitamins [31]. According to studies, Asian populations have switched away from plant-based foods and toward meat and animal products, resulting in a 75% increase in worldwide demand for meat products by 2050 [31,32,33]. This increased demand is problematic because present large-scale animal farming operations (which produce more than half of the world’s meat) are linked to public health hazards, environmental degradation, and animal welfare concerns [34]. Its growth is fueled by several concerns: (1) global population growth; (2) the environmental impacts of animal agriculture, such as land use, greenhouse gas emissions, and biodiversity impacts; (3) animal ethics, including farm animal living conditions and slaughter; and (4) the impacts of animal agriculture on human health, such as animal-borne diseases and antibiotic use [35,36].

Extreme weather events, shifting climate patterns, and rising temperatures are disrupting established agricultural systems, making populations around the world more vulnerable to food shortages. Future climatic variability, combined with population expansion, is expected to have a significant impact on global food security [37,38]. Heat stress, changing rainfall patterns, and resource limits all have an impact on livestock health and productivity, and the industry as a whole contributes significantly to greenhouse gas emissions. This results in a feedback loop in which food production suffers from and contributes to climate change [39,40,41].

**Figure 2:**
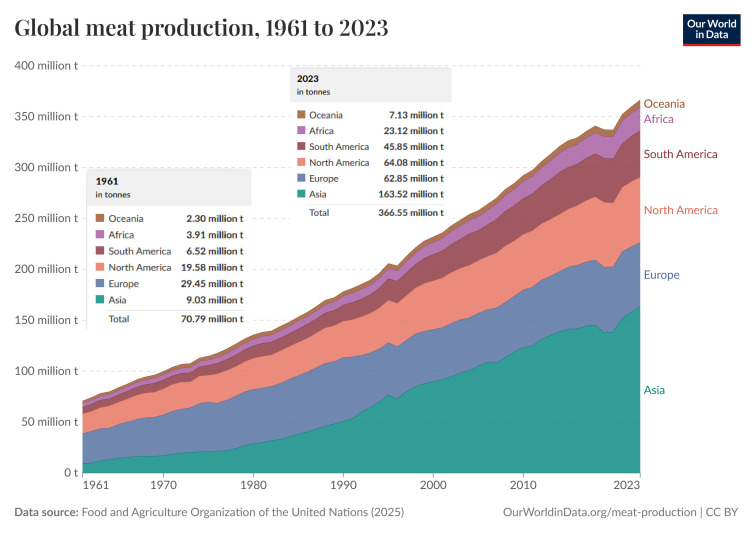
Global meat production, 1961 to 2023 [44].

The global food industry faces significant challenges in meeting the growing demand for meat while ensuring environmental sustainability and addressing animal welfare concerns [42,43]. Global meat production has increased five-fold over the last 60 years (Figure 2) [44].

Meat substitutes are being created (algal proteins, plant products, mycoproteins, and insects) [45]. Until recently, meat substitutes were plant-based products (mostly legumes and cereals) that were meant to mimic the sensory properties of animal-based diets [46,47]. Despite industrial progress in the production of plant-based foods and the diversity of products available on the market, these products continue to lack the nutritional, sensory, and technological characteristics of their counterparts, resulting in low acceptance [48,49]. Promoting the use of alternative proteins, such as plant proteins, insects, fungi, legumes, algae, or CM [50,51,52], it should be remembered that meat contains not only protein but also other minerals, energy, fatty acids, vitamins, and exogenous amino acids, which plants do not provide or have low bioavailability [53,54]. In this setting, and given the importance of food on human social connections, meat consumption habits, and meat’s distinct qualities, the hunt for alternatives with adequate technological, sensory, and nutritional properties is rising. Considering the foregoing, the manufacturing of CM is becoming an option [55,56,57]. Cellular agriculture and the creation of CM is one of the most significant technological achievements in food production [14,58,59]. A well-known example of cellular agriculture is cell-based food, which has been in development for almost two decades [60]. Concerns about the environment, animal welfare, public and consumer health related to livestock production, the use of antibiotics in the animal industry, and food safety are the main driving forces behind the production of CM [61]. CM is frequently portrayed as environmentally safer. Compared to traditional meat production, CM uses around 7–45% less energy, 78–96% fewer greenhouse gas emissions, 99% less land, and 82–96% less water, depending on the product [62,63,64,65]. The assumption of Tuomisto and Mattos [66] was that cyanobacteria would be used as a source of energy and nutrients. As a result, somewhat large industrial energy requirements have been estimated for CM [67]. Overall, the statistics indicate that CM production may emit greenhouse gases equal to pork and poultry production, but substantially less than beef production [68]. As a result, the environmental impact of CM is still debatable, and it may really be no better than that of traditional animal meat production systems [69]. The range of greenhouse gas emission reductions according to the LCA for CM production compared to conventional meat is estimated at 17–92% (depending on the energy mix—scenarios with renewable energy: reductions closer to the upper range, and in scenarios with fossil energy: minimal reductions or no advantage; Energy consumption: change: from +20% to −60% compared to conventional meat (some scenarios show higher energy consumption due to intensive process control); Models from 2023 indicate that optimization of bioreactors and cleaner energy shift the results towards reduction [70]. Land use—reduction: 63–95% (the most stable and least controversial parameter in the LCA of cultured meat) is mainly due to the lack of the need for animal breeding and feed crops. Water use—reduction: 51–78% (depending on the method of production of nutrient and energy components). In scenarios with intensive production of nutrient components, reductions may be closer to the lower range). Meat LCA are prospective analyses, based on models of future factories, not data from existing plants. The results strongly depend on the energy mix (RES vs. fossil fuels), bioreactor efficiency, composition and environmental cost of the medium, and assumptions regarding purification, sterilization, and cooling [70,71].

Finally, the ultimate goal of CM production is to create a product that differs significantly from previous meat mimics in terms of meat-likeness. According to research, people expect CM to be more meat-like than plant-based alternatives. To match customer expectations and attain market competitiveness, cultivating fat and other tissue components is essential [72].

In general, the main advantages of CM described in the literature stem from the perspective of animal welfare and the reduction in greenhouse gas emissions. Furthermore, the production of CM mitigates and prevents public health problems associated with large-scale livestock husbandry, such as zoonoses and antibiotic resistance [16,73,74,75]. Those involved in the production of CM emphasize the potential benefits of these products over their counterparts, such as reduced environmental damage, improved nutritional quality, the prospect of low product costs, and the possibility of large-scale production to meet population growth and, consequently, food demand [76,77,78,79,80].

Cellular agriculture has various market penetration obstacles, including technological, economic, safety, regulatory, and social issues, all of which have been updated several times since its inception [18,81,82,83,84]. Another factor to consider is that completely replacing traditional meat with CM may have a negative long-term impact on poor nations’ agriculture-based economies [85].

According to a Good Food Institute estimate, by 2030, the cost of manufacturing CM could be as low as USD 5.66/kg, comparable to conventional meat, in a model production facility with optimal production efficiency and appropriate finance techniques [86].

## 4. CM Quality

The distinctive flavor of meat is possibly the most difficult quality to identify, as it is composed of about 1000 water- or lipid-soluble molecules [87]. The texture of typical meat comes from the maturing period, which happens only after the animal has died. When oxygen is removed, lactic acid builds up and the pH drops, activating multiple enzymes necessary for protein breakdown and meat tenderization [32]. Changes in sugars, organic acids, peptides, free amino acids, and adenine nucleotides, as well as the production of taste precursors by peptides and postmortem interactions amongst breakdown products, all contribute to meat fragrance. When typical meat is heated, the Maillard reaction products, as well as the degradation of lipids, peptides, and amino acids, and the interactions between these resultant molecules, all contribute to meat fragrance [18,88,89,90].

Quality assessment of food goods, particularly meat, is critical in giving objective information about their properties. However, in the case of cell-based food items, the availability of scientific evidence for quality assessment is significantly limited when compared to regular beef Nutritional value is the most important aspect of food and a primary motivator for consumer intake. As a result, many studies on CM have evaluated its nutritional value from a variety of angles, including moisture, protein, and fat content [19]. However, product acceptance is largely determined by sensory appeal. In the case of CM, it is unclear to what extent flavor precursors will be present and will react with other degradation products, as these flavor precursors are formed postmortem in conventional meat [18]. It is worth emphasizing that many of the chemicals that accumulate in muscle are not produced by the muscle itself, but rather from animal feed materials digested and changed by non-muscle organs. Unless these compounds are given to the culture media and absorbed by the cells, they will not be present in the CM, impacting processes that determine flavor, texture, color, and nutritional features [18].

Electronic tongue analysis, visual appearance rating, and sensory tastings were used to determine the similarity to conventional meat and consumer preference. The appearance, form, and color of CM were evaluated by grilling or frying it [91], and pigments were utilized to duplicate the meat color [92,93]. The color of cultured fat was shown to shift towards a more prominent yellow hue with extended culture durations [94]. The tasting panel concluded that the lack of fat made the burger slightly dry, but no in-depth quality or sensory evaluation was performed. The only other, modest, sensory test on cultured cells reported in the scientific literature dates from the early years of CM experiments and involved smelling and observation, but no tasting [18]. There were considerable changes in amino acid composition between laboratory-grown beef and its natural counterparts, with the exception of valine and tyrosine [10]. Furthermore, fat content investigations have demonstrated a shift in the fatty acid composition of beef subcutaneous adipose tissue toward the later stage of culture, rather than the earlier stage [94]. Umami, one of meat’s flavor qualities, is connected with nucleic acid molecules. Analysis of nucleic acid components revealed that CM had substantially lower quantities than natural meat. As a result, electronic tongue analysis indicated significant variations from traditional meat profiles [10]. Sensory evaluations based on actual human meat intake have confirmed these findings, demonstrating that cultured muscle tissue provides better initial taste impressions than natural beef [95]. These findings imply that CM provides a fascinating sensory experience to sensory test participants, raising the prospect that CM could be a suitable substitute for regular meat [96]. Fat also influences the flavor, texture, nutritional value, and visual attractiveness of meat. Despite progress in meat analogue production procedures, replicating the sensory qualities of fat remains a problem [97], particularly because the flavor of traditional meat is regulated by aging, a critical process that has yet to be investigated in CM. Heating fungal protein hydrolysates or combining soy sauce hydrolysates with defatted soy, for example, produces beef-like aroma compounds that can be employed to improve the meaty flavor of cell culture-grown tissue [81]. Other non-protein substances can be improved by altering the culture medium. For example, by adjusting the lipid composition in the medium, the ratio of saturated to polyunsaturated fatty acids in CM can be better managed, although caution should be exercised due to the potential impact on rancidity [16,73].

## 5. CM Production and Quality Engineering Towards Functional Foods

CM production is based on the in vitro development and differentiation of myocytes and muscle fibers and requires optimal culture conditions. Therefore, it is necessary to identify culture conditions that maximize the proliferation and differentiation of muscle satellite cells (MSCs) into myotubes and muscle fibers while maintaining a texture and flavor comparable to that in conventional meat. Medium, pH, temperature, and muscle type are among variables that influence MSC proliferation. Studies on pigs, turkeys, and chickens show that culture temperature affects the ability of muscle-specific MSCs to proliferate and differentiate differently [98]. One potential future CM technology is the use of induced pluripotent stem cells (iPSCs) produced from adult cells rather than embryonic stem cells [99].

Continued study and development are required to fully understand the media components and their significance in increasing culture efficiency in CM production. Meeting all the desired conditions to optimize cell proliferation and differentiation remains challenging [100].

Fat-muscle co-culture (CM) is a novel high-tech food production process that involves co-culturing myocytes and adipocytes on a porous scaffold [101,102,103]. This method of meat culture provides numerous advantages, the most notable of which is that it improves the sensory and nutritional properties of the finished product. One notable advantage is the capacity to duplicate the complex structure of animal meat, such as muscle fibers, connective tissue, and fat, thereby increasing the texture and flavor of the CM [104]. Co-culturing different cell types presents various obstacles, most notably the intricacy and unpredictability of cell–cell interactions. One key challenge is effectively predicting and controlling cell-to-cell interactions [105]. This form of culture has certain restrictions that must be addressed in order to achieve successful industrial-scale manufacturing. One of the primary constraints is the high cost of cell culture media and growth regulators, as well as the requirement for a highly skilled personnel, which has a substantial impact on the entire cost of in vitro meat production [106]. To solve co-culture’s shortcomings, future research should focus on the utilization of integrated co-culture systems incorporating several cell types. Furthermore, research into the development of culture media and growth regulators for co-culture procedures is critical in lowering the costs involved with CM production [107].

CM is designed to have the same appearance, taste, texture, and nutritional value as traditional meat [108]. Food processing biotechnology allows for the creation of CM that preserves its fresh color and appearance. CM can be improved in flavor and texture by controlling the quantity of fat in the meat with bioprocessing technologies, and the addition of protein has allowed CM to have a color comparable to traditional meat [109]. Although the resulting meat is pink rather than the typical blood-red product found in conventional beef, some people who are put off by the presence of blood in meat may prefer CM [110,111].

Initial research mostly used extrusion-based bioprinting to create thin films or small structures, thereby proving the practicality of this method [112,113,114]. However, these early models lacked the structural complexity and scale required to replicate real meat. Currently, modification of meat-like tissue by integrating muscle, fat, and vascular tissue using bioprinting has produced a more realistic muscle-like structure [115] in vitro cell culture [116], designing a hydrogel structure to align muscle fiber orientation [117], and assembling a structure made of highly differentiated muscle and adipocytes to improve fat distribution in CM [100]. Although substantial progress has been made in employing 3D printing to improve the quality of CM, it still falls short of entirely replicating real meat’s sensory qualities and nutritional composition. Recently, 4D printing technology has expanded the field of CM. Its dynamic modulation capability has huge potential for improving the look, texture, color, flavor, and nutritional value of CM [118,119]. The myofibrillar structure stores water based on the pH shift caused by post-harvest anaerobic metabolism. This illustrates the necessity of maintaining myofibril formation in cell culture, assuring lipid supply to the product, and maintaining an optimal pH profile after harvest, all of which influence final flavor and juiciness [120]. Recently, methods for stabilizing vegetable oils in the liquid phase have been highlighted, including pre-emulsification, microencapsulation, and the production of oleo-gels [121,122].

Bioprinting is the process of extruding bioinks into a hydrogel matrix to efficiently fabricate complex, multi-component structures with improved structural integrity [123]. The supporting hydrogel stabilizes the bioinks, making it possible to create intricate designs that closely resemble real meat structures. Three-dimensional bioprinting, which engineers muscle and adipose tissue in regulated configurations, has the ability to closely imitate the natural structure and composition of conventional meat. Furthermore, by changing the bioink composition and printing conditions, it is possible to fine-tune the flavor, nutritional profile, texture, and structure of CM [124].

The medium used to cultivate meat in vitro is critical to its success. Furthermore, the selection of cultures manipulates and influences flavor and fatty acid composition, allowing raw CM to be adjusted to nutritional needs. These are sometimes referred to as designer meats, and they also provide health benefits due to the presence of specific vitamins [73]. For example, coculture allows for the personalization of fat-enriched beef. Adipocytes produce fat. Consumers enjoy the trend of supplementing meat with other flavors, while the introduction of nutritionally engineered foods has piqued stakeholders’ interest in specific nutritional properties [32,73].

Although in vitro generation of CM is in the early phases of development, it has the potential to be considered a functional food due to several factors, such as controlled nutrient composition, health-enhancing additives, reduced harmful ingredients, and tailored bioactive compounds [125]. The advantage of engineered meat is its production in bioreactors, which allows for the adjustment of the nutrients added to the medium. Such production requires significantly less space compared to livestock production and animal feed. The strategic purpose of these biologically engineered food products is significant because they can be easily transported to supply military camps on war fronts in polar regions and harsh environments, where providing nutritious food becomes a challenge. Similarly, they can be fed to astronauts, providing a healthy and sufficient source of energy. Furthermore, they have been employed as nourishment for research stations located in oceans and high latitudes, where protein and fat-rich diets are required. Thus, in vitro meat has a significant advantage in satisfying food needs in emergency situations while also assuring long-term survival [32]. CM can be genetically modified to meet specific dietary needs, making it an invaluable resource for individuals with dietary restrictions, allergies, or conditions such as malnutrition. Furthermore, its ability to provide high-quality protein and essential nutrients could provide a viable solution for patients recovering from surgery or struggling with chronic illnesses. These advantages of custom-grown CM are features that position them as potential functional foods of the future, capable of providing specific health benefits when incorporated into the human diet [125]. Embedded bioprinting also enables for fine-tuning of mechanical properties by including multiple biomaterials, theoretically producing textures and mouthfeel comparable to normal meat [126,127].

The majority of chemical metabolites found in typical meat are not just derived from muscle but also from the animal’s diet and biological metabolism. These, together with the interaction of proteins, lipids, carbohydrates, neurons, and blood vessels, contribute to the distinct flavor of meat [32]. At the same time, it is crucial to note that flavor is heavily influenced by changes in sugars, organic acids, peptides, free amino acids, and breakdown products that occur only after slaughter [16,128]. Culturing animal cells on vegetables is resulting in a new class of hybrid foods that contain a healthy balance of animal and plant elements. The veggies we choose, from Chinese chives and shiitake mushrooms to luffa, are low in calories and high in vitamins, minerals, and antioxidant carotenoids [129]. This considerably improves the nutritional value of genetically modified beef and may bring health benefits such as reduced risk of obesity [130], colorectal cancer [131], diabetes [132], and so on. Mushrooms also contain glutamate [133], which contributes to their umami flavor, which is similar to that of actual flesh [134]. Previous research has used by-products such as grape peel [135] and raspberry pomace [136] in a smart composite matrix film to display visible color changes in response to pH variations across a wide range of grades from 2 to 13, in order to check product freshness. This color change, which normally occurs from a light to a darker color shade, clearly indicates the freshness level of the packed product. The efficiency of pH-responsive color changes is determined by the film’s material composition, which must be constructed so that customers can easily detect the changes. Furthermore, a dual-action packaging film, called innovation packaging, was previously developed using agro-food by-streams, allowing interaction with the package contents to change state (active packaging) and real-time monitoring of food freshness to communicate with the consumer (smart packaging) [137].

In meat production, these cells are often seeded onto edible scaffolds that mimic the properties of muscle tissue’s extracellular matrix (ECM) [58,138,139]. Three-dimensionally printed scaffolds or electrospun polymer fibers coated with extracellular matrix proteins, such as collagen, can increase adhesion by enhancing media circulation and nutrient transport [140]. The scaffolds can then be cultured in bioreactors and provided with growth media in controlled environments [20], which support both cell growth and tissue maturation, ultimately developing into meat [141,142,143]. Replicating the texture, flavor, and nutritional profile of conventional meat remains a significant hurdle. A key factor in overcoming this challenge is the development of three-dimensional (3D) edible scaffolds that mimic the extracellular matrix (ECM) during cell growth, proliferation, and differentiation [116,144,145]. Scaffolds are used to guide the development into a 3D structure during the cell growth phase. This is critical for cells to adhere, proliferate, and differentiate, while maintaining optimal porosity to enable nutrient and oxygen delivery to the cells [146]. Depending on the scaffold used, cells may require removal from the scaffold or the scaffold may be used together with cellular biomass to produce a meat product [95,147,148]. Biomaterials considered for scaffolds in CM production are presented in Table 1.

Based on medical experience, synthetic materials have begun to be used as CM scaffolds. However, the use of a polystyrene scaffold, for example, requires its removal as it is not suitable for consumption. Scaffold removal can be accomplished in a variety of ways, including mechanical removal of cells, enzymatic detachment of cells from the scaffold, or the use of a scaffold material with modified properties, such as increased temperature or pH, where a change in temperature or pH causes a reversible change in the scaffold structure, allowing cell detachment [141,148]. However, synthetic scaffolds can produce harmful byproducts and animal-derived materials, so for safety reasons, researchers are focusing on finding and improving natural scaffold materials. Researchers have used edible scaffold sources such as chitosan, alginate, collagen, or gelatin [11,95,191]. Collagen and other animal-derived polymers are regarded as ideal options for CM creation. However, collagen can have a negative impact on the essential amino acid composition of CM due to an increase in non-essential glycine [17,146]. The disadvantage of animal-derived materials such as collagen and gelatin, although they are harmless, is their often high price and non-renewability [25,192]. Biopolymer hydrogels must have the right physicochemical qualities to work as scaffolds for CM, such as mechanical properties, fluid binding, and permeability [193]. This has resulted in a trend toward renewable plant-based materials that provide sustainability, biocompatibility, and the capacity to mimic the structure of traditional meat. Examples include plant proteins [170,191,194,195] plant polysaccharides [117], plant leaf veins [196,197,198], and plant composites [199]. Non-mammalian scaffolds (e.g., plant or algae-based) are gaining popularity as possibly stable, low-cost or cost-effective alternatives that are edible and biodegradable [146]. Other alternative scaffold materials include polysaccharides (chitosan, cellulose, and alginate), complex composites (lignin or textured plant protein), and plant proteins such as zein (corn), wheat glutenin, pea, soy, and algae [140,194,200]. There are still issues with plant-derived scaffolds for CM despite a lot of research, such as their low mechanical strength and requirement for chemical processing. Additionally, even while aligned scaffolds have been demonstrated to enhance muscle development and maturation in comparison to unaligned structures [201], it is still very difficult to accurately replicate the intricate microstructure of muscle tissue. The organoleptic characteristics of traditional meat, including appearance, taste, texture, structure, and mouthfeel, should be demonstrated by CM scaffolds [202,203]. Because natural materials are more palatable, biocompatible, and biodegradable, they are more suited for building the scaffolds needed to produce CM [202]. When included into a finished product, such hybrid meat products, the scaffold needs to be safe and edible [192]. Alginate, one of the most prevalent natural and synthetic biomaterials worldwide, is frequently used to create low-cost tissue scaffolds [204,205]. Growing cells can be attached to scaffolds or microcarriers, which act as a structural support network for tissue development. In addition to their primary function, scaffolds carry functional components or bioactive compounds in CM that may be beneficial to health. Edible scaffolds made from various edible polymers can be used to generate solid structures or coatings that gradually release nutrients or functional compounds during digestion [138,206,207,208,209]. When included into the finished product, these scaffolds—which can be protein-based, like collagen—can have a direct impact on the amino acid composition [17,194]. Although the essential amino acid profile of meat in vitro may be adversely affected because collagen has a larger proportion of the non-essential amino acid glycine, animal-derived polymers like collagen are thought to be very suited for the development of CM [146]. Edible scaffolds or microcarriers can be integrated into the finished product. In contrast to earlier methods, the use of edible microcarriers or scaffolds as a food ingredient or additive in the manufacture of CM requires compliance with regulations. These products primarily contain the following edible polymers: (i) polysaccharides like starch, alginate, carrageenan, chitosan, cellulose, carboxymethylcellulose, and pectin; (ii) polypeptides like collagen, gelatin, and gluten; (iii) paraffin and shellac, and their compounds/synthetics like polyethylene glycol (PEG) and cross-linked polygalacturonic acid (PGA). In the food sector, these materials are frequently employed as emulsifiers, thickeners, coatings, and stabilizers. In this instance, the edible polymer employed as a cell substrate during cell proliferation can be engineered to improve or add desired features like texture, flavor, or color, and the dissociation stage can be completely skipped [57].

Several innovative studies have been published using techniques such as plant protein scaffolds [210], 3D printing [194], and hydrogels [211] in the field of cultured fats. Crucially, a number of studies have shown a link between dietary fat, namely fat amount and quality, and the risk of metabolic disorders [212,213]. The precise connection between dietary cholesterol levels, the risk of cardiovascular disease, and numerous other disorders has been clarified by earlier research [212]. Crucially, a number of illnesses, including as arthritis, cancer, and cardiovascular disease, are significantly influenced by the dietary N-6 to N-3 fatty acid ratio. All of this has brought attention to the necessity of meat products that are functionally optimized. By altering the lipid composition of the media, CM enables nutritional modifications that are not achievable with traditional animal breeding, such as adjusting the ratio of polyunsaturated fatty acids (PUFA) to saturated fatty acids (SFA) [16]. It has also been demonstrated that adjusting the composition of the media can help produce cultured beef that is high in oleic acid [214,215].

Supplementation of plant proteins to CM scaffolds provides a nutritious protein source [159,194]. Edible plant resources, such as decellularized spinach, wheat glutenin, peanuts, and others, have been the primary focus of research on solid-structure scaffolds [170,175,194,200,210,216]. Developing scaffolds based mostly on plant proteins as low-cost, sustainable biomaterials for tissue engineering applications [217,218] and CM [58,219] has also drawn more attention in recent years. Extrusion, heat treatment, salt leaching, or decellularization of plant tissue using the plant structure can all be used to create porous scaffolds [107,220]. Decellularization based on plants and fungi has so garnered a lot of attention. Advantages of plants and fungi include scalability, cost-effectiveness, quick development, and ease of culture [178,221,222,223,224]. Myoblast culture and subsequent differentiation on decellularized fungal scaffolds has demonstrated the successful creation of muscle tissue constructs [225,226]. Conversely, the biomass buildup, ease of growing, and low re-source requirements of fungus, particularly edible mushrooms, make them a desirable source for scaffold fabrication [224].

CM is looking for technological advancements in the following areas to fulfill its promises: big, intelligent bioreactors [142,227], high-quality muscle and adipose tissue [88,96,228], low-cost medium, and functional scaffolds. Natural plant scaffolds are renewable and can be engineered to break down at a rate that is ideal for tissue growth [140].

## 6. Shaping the Quality of CM

Nowadays, a large number of businesses, mostly startups, are creating and manufacturing early-stage CM products with an emphasis on muscle cell development. However, before a finished product with qualities comparable to traditional meat can be produced, a number of factors need to be taken into account, including nutritional value, food safety, ethics, organoleptic features, production scale, and costs [17,25,73]. As a result, the methods utilized in cell-based food, which are primarily derived from the medical fields of tissue engineering and cell culture, need to be improved [19]. Adherent cell suspension culture and three-dimensional edible scaffolds are only two examples of the numerous new technologies and innovations that have emerged as a result of the rapid technological and industrial development of CM in recent years. The industrialization of CM currently faces three major obstacles: lowering costs through technology and equipment, enhancing regulatory communication, and boosting consumer outreach and awareness [229]. Examples of activities aimed at modifying the quality of CM are presented in Table 2.

### 6.1. Shaping the Chemical Composition and Nutritional Value Towards Functional Foods

Currently, most CM tissues consist solely of muscle tissue. Ground CM, consisting of muscle and fat, is produced by separately culturing muscle fibers and adipose organoids, which are then combined to create the final CM product. To cover the entire range of livestock meat production, full-thickness tissues (i.e., steaks) must be engineered, and therefore, more advanced tissue engineering approaches are needed [82,170].

Increasing the protein content of CM can be achieved by various strategies. (I) Sarcomere synthesis is induced by electrical stimulation. Despite its great effectiveness, this approach is expensive, making it unsuitable for widespread use [17]. (II) Optimization of the culture medium by providing a higher content of free amino acids and resulting in a higher protein content. However, as claimed Broucke et al. [16], even though this strategy might be more economical, more research is required to understand how cells absorb nutrients and what happens to them after internalization. (III) Using protein scaffolds that are edible or biodegradable. This option would allow for the modification of the amino acid composition of cultured products in addition to being cost-effective and widely applicable. More precisely, matrices high in essential amino acids can be chosen for the creation of these structures, either by employing genetic engineering to create transgenic organisms that can synthesize the desired amino acids or by choosing derivatives obtained from plants [16,128].

Fat significantly influences meat quality, influencing flavor, aroma, and tenderness, along with protein content [240]. Age, diet, and habitat all have an impact on the amount and makeup of fat and fatty acids in meat, which differs between species and breeds within species [97]. It is essential to directly interfere with cultured cells, especially fat cells. According to Broucke et al. [16] and Fraeye et al. [18], fat is actually essential for the final product’s aroma, juiciness, and tenderness. Co-cultures of muscle cells and adipocytes, the use of preadipocytes to increase intra-muscular adipose tissue [18,101], the addition of carotenoids, which can prevent fatty acid oxidation by limiting their rancidity and preserving the final flavor [16,241], and the selection of a biomaterial that permits the differentiation of a particular cell type, such as adipocytes [82] are some possible solutions. Lastly, it is feasible to directly incorporate flavors that reflect customer preferences into the finished product. According to Zhang et al. [81], heating potential substitutes like soy sauce hydrolysates, defatted soy protein, or mushroom protein results in taste molecules that resemble those found in beef.

In an effort to mimic these properties of fat in CM, an oleogel-based fat substitute (FS) was developed. A combination of direct and indirect methods was used for its production. Oil droplets were structured with glycerol monostearate (GMS) in an aqueous protein solution using an emulsification procedure followed by freeze-drying [242]. Some compounds, such as phenolic compounds derived from sources such as olive leaves, which enhance the bioavailability of essential minerals in poultry meat production, may be useful for developing a culture medium for in vitro culture of chicken muscle cells [243].

Because the final product (CM) may have a different nutritional profile than the product it replaces (conventional meat), nutritional hazards have been brought to light [93,244]. Certain elements found in normal beef are absent from CM until they are provided, such as iron, creatine, and vitamins B_12_ and D, which are delivered to muscle cells rather than produced there [57]. To achieve this, it is necessary to add these nutrients directly to the protein binding and transport medium to facilitate cellular uptake [16]. Additionally, it is unknown if the vitamins and minerals found in CM and potentially added through culture medium will have the same beneficial impact on human health [73].

The final CM’s nutritional characteristics are significantly impacted by how natural plant fiber (NPF) scaffolds control cell development. Moreover, meat’s amino acid makeup plays a critical role in determining its taste perceptions, which are intimately linked to the range of fundamental flavors. These include umami, sourness, sweetness, saltiness, and bitterness, all of which add to the intricate flavor experiences of meat [10]. The size of the scaffolds used to produce cultured muscle cells may be greater than that of the muscle cells themselves, which could lead to a decrease in nutritional density, in contrast to traditional meat from farm animals [18]. Enhancing the nutritional profile of CM could also be accomplished by genetic alteration of animal cells. For instance, Stout et al. [241] showed how prokaryotic enzymes may be engineered into primary bovine muscle cells and immortalized mouse muscle cells to produce non-native carotenoids (phytoene, lycopene, and β-carotene).

### 6.2. Shaping Sensory Quality

Getting a meat product with enhanced sensory qualities, like texture, color, and flavor, from alternative protein sources is the largest challenge [245,246]. Cell isolation, cell culture, differentiation, and tissue development are the four stages of CM manufacturing that result in a traditional meat-like product [247]. However, umami and bitter flavors may be less prominent, as found for CM obtained from chicken and bovine muscles [10], even though instrumental analysis of CM has showed comparable textural features to normal meat [248].

Myoglobin and iron concentrations are the two fundamental factors that determine the color of the traditional product [249]. The lack of myoglobin, which is repressed by the cultured cells in the presence of oxygen, and the low concentrations of iron in the primary culture media are the two reasons why laboratory-grown muscle fibers often appear yellow [249]. By reducing oxygen levels, raising the iron content in the culture medium, and directly adding natural colors to the finished product, myoglobin production can be stimulated to provide the typical meat color [18]. According to Zhang et al. [81], adding hemoglobin straight to the culture is another potential remedy. However, this solution would necessitate the costly and time-consuming extraction of hemoglobin from animal blood, plant tissue, or microbial tissue, making it impractical on a wide scale [81].

The absence of the protein myoglobin, which gives animal meat its red hue, is one of the issues with CM. Hemoglobin extracted from animal blood or its derivatives, as well as natural pigments like sugar beet or saffron, can be utilized in the meat production process to solve this problem [250]. Meat analogs can be colored to resemble real meat by adding natural colors or dyes. For instance, in order to give CM products a crimson hue resembling that of raw beef, scientists have added proan-thocyanidins [251].

Color and appearance have a significant impact on consumer acceptability. Due to the very low myoglobin content, CM is almost colorless. Direct addition of myoglobin or hemoglobin to the medium, the use of scaffolds seeded with muscle cells to produce more attractive CM, or the addition of colorants such as beetroot juice and saffron during post-processing have been explored, although the latter can also alter the flavor [18,81,85]. Naturally occurring meat nanofibers influence the texture and color of meat after cooking. Therefore, the use of nanotechnology to produce CM may prove effective. Furthermore, the packaging of meat products makes significant use of nanotechnology [252]. Replicating the intricate structure and texture of real flesh is a major technological challenge. Each of the components of natural meat—muscle fibers, fat cells, and connective tissues—contributes to its structure, texture, flavor, and mouthfeel, all of which are important aspects of consumer acceptance [253,254]. When sterilizing materials, autoclaving imparts biomimetic stiffness to vegetables, similar to that of animal tissues. Adjustable autoclaving time provides vegetables with mechanical tunability while maintaining structural integrity [227]. In CM products, intramuscular fat is crucial to achieving the right meat flavor, juiciness, and softness [255].

Meat’s ability to retain water affects sensory factors and the performance of the finished product. The degree of ac-tomyosin complex formed in cultivated cells will impact their ability to store water, just like in traditional meat products [18]. However, this can have a substantial effect on water-holding capacity because cultured cells mostly possess embryonic or neonatal actin and myosin [17]. Using mechanical processing, seasonings, and food additives, post-harvest processing enhances the texture, flavor, and appearance of CM [159]. While enhancing microbiological safety, methods like high-pressure processing and precision flavor engineering assist mimic the sensory qualities of traditional meat [256,257]. A recent study measured the palatability of small amounts of cultured muscle tissue (CMT) using an electronic tongue system and discovered that CMT had lower levels of umami and bitterness intensity than traditional meat [10]. As a result, it is now feasible to investigate methods for optimizing the generation of muscle satellite cells (MSCs) so that the cells have flavor qualities similar to those of traditional meat [98].

### 6.3. Shaping CM Safety

CM is currently comparable to regular meat because of advancements in microbiology and chemistry, and it is thought to be ethical, healthy, nutrient-rich, and environmentally benign. Despite its success, the use of materials in the development of synthetic meat presents a number of difficulties, necessitating safety evaluations and regulatory frameworks to control the hazards involved in the manufacturing of CM [32].

CM can be formed into a tissue structure using 3D [11]. Bioprinting arranges cellular and acellular components “to construct complex functional 3D living tissues”, starting with CM production and ending with printing “muscle cells, fat cells, and extracellular matrix supporting cells” [258]. In addition to regulating the fat content of CM, the ability to enrich CM with essential nutrients, such as iron and vitamin B_12_, could effectively alleviate common nutritional deficiencies [32,73,125].

Cross-contamination or unintentional introduction of cells or microbes into the culture system can infect cell lines. This has the potential to negatively impact or perhaps destroy the culture’s performance. When a fast-growing microbe takes over the culture, devouring its resources and competing with the chosen cell line, bacterial, fungal, or yeast contamination (or infection) may result [57].

## 7. Consumer Preferences Towards CM

Consumers choose a product based on the attributes they consider most important. Intrinsic attributes physically differentiate the product, while extrinsic features enhance the perception of the value it provides. The most valued attributes of beef are color, aroma, expiration date, price, production date, and tax inspection stamp. The most valued benefits are related to sensory aspects: freshness, flavor, tenderness, leanness, and juiciness [259,260].

Public awareness, perceived naturalness, perceptions of food risk and food neophobia, ethical and environmental issues, and worries about food safety and human health are some of these [261,262,263,264]. However, consumer acceptance is the biggest obstacle to CM’s commercialization. Consumer attitudes are a major factor in the adoption of novel food technology, as researchers have already shown [265]. The findings indicate that consumers have mixed feelings about consuming CM. While many respondents would eat farm-raised meat, the majority would still choose traditional meat [261]; according to other studies, there is a strong readiness to eat CM [265,266] or at least attempt.

Studies have shown that between 19% and 66.4% of people are willing to taste cell-cultured beef. However, people are often less inclined to frequently buy CM or use it in place of traditional meat. We must interpret readiness to try and consume the product cautiously because public awareness of it is still quite low. Additionally, the adoption of alternatives, like plant-based meat substitutes, is frequently contrasted with the possibility for consumer acceptance of cultured proteins [51,263,267]. Customers view an excellent meat protein substitute as one that satisfies their dietary requirements while closely mimicking the sensory and functional qualities of conventional meat [8]. One of the main obstacles to consumer taste and desire to try CM, particularly among meat eaters, is its poor flavor quality, which is linked to the absence of meat flavor [268,269]. Customers looking for healthier meat products are reportedly unwilling to sacrifice sensory quality [270,271]. A lack of knowledge about the technology and its advantages is frequently cited as the reason why consumers are skeptical about new food production systems. However, a distinction can be made between awareness and knowledge of the innovation, although in many reviewed articles, these terms were used interchangeably, and such a distinction is less common. Accepting new technology frequently starts with awareness (and familiarity). An attitude toward the new technology is not always the result of awareness; rather, awareness fosters curiosity and inclination [272].

Consumers in the Netherlands, Italy, Germany, the United States, Switzerland, Belgium, the United Kingdom, Spain, Brazil, Finland, and the Dominican Republic prefer plant-based alternatives over CM, according to studies on the acceptance of cultured protein in comparison to other forms of alternative protein (such as legumes, algae, insects, and plant-based meat substitutes) [263,273,274,275,276,277,278]. Compared to consumers in less developed nations, consumers in economically developed nations are typically more receptive to meat substitutes [278]. A study comparing China and India with the United States [279] found higher levels of acceptance in China and India, while French consumers showed lower levels of acceptance than consumers in other European countries because they considered CM to be unnatural and disgusting [262,280]. Price was a significant factor in readiness to buy, according to a consumer survey involving participants from Germany, France, and Africa. The majority of participants were unwilling to spend more for CM than for normal beef [281,282,283]. The acceptance of CM is influenced by sensory perception in addition to regional variations in awareness. Curiosity regarding CM’s organoleptic qualities, like taste and texture, is said to be the main reason people want to try it. Whether or not CM tastes like regular meat is a key factor in determining whether or not people will eat it [284]. Sensory quality is, in fact, a key factor in boosting customer acceptance [62,147]. However, in the near and far future, cultured beef can be made more palatable by modifying its taste and texture [285]. The taste of CM is perceived differently than that of traditional meat [286].

Recent findings suggest that acceptance and willingness to consume alternative protein sources may be increasing, particularly among young adults (aged 18–34 years) [287,288]. In general, younger, better educated meat eaters are more inclined to embrace CM [285,289,290]. Only half are willing to pay extra for CM, but the majority are eager to try it, purchase it frequently, or even use it in place of traditional meat [290]. Additionally, research have indicated that CM is more widely accepted than comparable food technology advancements like insect protein or genetically modified organisms (GMOs). Nonetheless, business viability depends on consumer acceptance, and methods to boost it are required. In order to accomplish this, efforts are concentrated on cutting expenses while maintaining the taste, texture, and look of conventional beef [36,285]. For instance, proponents of CM are creating plans to make the meat more palatable by using science and technology to enhance production methods and sensory qualities; increasing public awareness through behavioral science research; disseminating scientific data; and enacting legislative changes [291]. One of the best indicators of acceptance of CM has been shown to be prior knowledge of it [279,292]. Therefore, positive information and framing that elicit more favorable associations might lead to improved acceptance [285], such as labeling that highlights the product’s environmental or health benefits. The advantages that marketers choose to highlight, how the idea is covered by the media, and the features of the product itself all influence consumer acceptance of CM [293]. Since CM would be a novel product on customers’ tables, it is hypothesized that the more similar it is to conventional meat in terms of sensory and nutritional qualities, the simpler it will be to overcome food neophobia and even adhere to societal conventions and rituals [49].

Food neophobia, which has its roots in risk perception and society, explains why people are reluctant to eat unfamiliar foods [294]. Consumer acceptance of meat alternatives is thought to be hampered by food neophobia [261,295]. Neophobia has been found to be a significant contributing factor to the low acceptance of CM in a number of consumer acceptance studies [296,297]. On the other hand, consumers who are informed and familiar with CM production may also reject it due to health and safety concerns, especially when they discover that CM is produced with hormones, growth factors, or other chemicals [73]. Customers are skeptical and even disgusted by the use of chemicals and synthetic ingredients in cultured beef [298]. Customers are less likely to accept CM since it is grown in a lab, which contrasts with how regular meat is collected and raises questions about its unnaturalness [299,300]. Electronic tongue research has also revealed that CM contains less umami, bitterness, and sourness than traditional animal flesh [10]. This analytical system indicates that CM lacks the flavor intensity of animal meat, which may result in a lower flavor quality for customers, even though it cannot accurately reflect human sensory assessments.

According to the research, flexitarians seem to be a perfect target market for meat substitutes because, despite their continued meat desires, they are receptive to different diets [49]. This trend toward less meat eating is being driven by financial, health, and environmental advantages [301]. But it is crucial to remember that this is not always the case because flexitarians differ from meat eaters as well as from one another [302].

Due to customers’ dietary habits and ideological perspectives, food choices are greatly fragmented. As a result, it is unreasonable to anticipate the launch of novel food items that appeal to every consumer group [303]. Consumer perception of CM is currently influenced by many factors, such as ethical values, nutritional value of the meat, political views, level of education, age, socioeconomic factors, and product knowledge [252]. The majority of the population is willing to consume CM, despite numerous opposing views on it in the current context [289]. Consumers have various concerns for many reasons. These include the perception of CM as unnatural, resulting from labeling it “in vitro”, “synthetic”, or “laboratory-grown” [304], and the fear of the disappearance of various rituals (e.g., Thanksgiving turkeys) [305]. Additionally, its market application is currently limited by nutritional, technological–functional, and sensory challenges, a lack of regulatory guidance, high costs, scaling challenges, public perception (such as public neophobia and technophobia), and a lack of knowledge about potential health benefits or food safety risks [16]. The growth of the meat market depends on consumer acceptability [261]. These days, a lot of consumers show hostility to CM because they are worried about new food products and technologies. Conservative worldviews, nature bias, speciesism, social dominance orientation, and mistrust of science are the main factors influencing their opinions [50]. More precisely, Etter et al. [306] came to the conclusion that cultured beef, chicken, and pork are not as popular as any other traditional or alternative protein source; in fact, they are even less popular than insects. The acceptability of these three proteins is not significantly different between cultured chicken and beef, but it is much lower for cultured pork [307]. Meat consumption has not significantly decreased in certain developed nations, which can be explained by opposition to the idea of lowering individual meat consumption and the belief that individual meat consumption has little bearing on the general context of climate change [308,309]. However, in other countries, the decline in meat consumption appears to be more significant [308,310,311] or there have been changes in the type of meat consumed, mainly for price and health reasons [312,313]. However, due to environmental concerns and demographic factors, customers in Western Europe, China, and Germany typically have favorable attitudes toward CM [314,315].

CM is a promising technology, but it is still in its infancy, and its industrial production faces many obstacles. These include consumer perception, the meat’s nutritional structure, and high production costs. According to research, the three most important factors influencing consumer perception of CM are price, texture, and taste [15,87,316]. In the case of CM [281] and meat analogs, the sense of naturalness is crucial because the greater the view of this product as unnatural, the lower its acceptability [262,266,317] and willingness to try [281]. Additionally, naturalness is linked to health, distaste [262,318], and perhaps the idea that this meat substitute is not true [263]. However, due to a number of advantages, such as reducing animal suffering [319], providing protein to low-income populations, and improving animal welfare, it has great potential in the future [289]. Consumer perception of CM is currently influenced by many factors, such as ethical values, the nutritional value of the meat, political views, education level, age, socioeconomic factors, and product knowledge [252]. Customers are become more conscious of the capacity market’s potential advantages in terms of lessening its negative effects on the environment, animal resources, and cattle welfare, as well as decreasing threats to public health [305,320]. It is generally accepted that young people—Gen Z, millennials, libertarians, flexitarians, and urbanites—are overrepresented among groups with a strong interest in CM [320].

## 8. Ethical Aspects of CM Production

Global sustainability still faces challenges despite the meat and processing industries’ efforts to adjust to society concerns about climate change, environmental effect, animal welfare, ethical issues, food safety, and overall quality [321,322]. By eliminating food waste and applying circular economy concepts, the objective is to achieve better efficiency while ensuring sustainable patterns of production and consumption. The global food system is moving toward more sustainable food production and innovation as a result of these issues [323,324,325,326,327,328]. There are also many worries regarding the care and health of farm animals as well as the lack of animal mistreatment. Recommendations to restrict or totally remove animal products from the diet [329,330,331] were prompted by negative views of livestock farming [332,333].

The possibility of greenhouse gas emissions and better environmental protection is another problem. Comments on how livestock affect the ecosystem may be dubious, yet they cannot be disregarded [334]. Monocultures and the energy required to produce CM could be dangerous.

Over the past 10 years, CM technology has drawn a lot of attention due to its potential to lessen the environmental impact of traditional agriculture and do away with the necessity for animal sacrifice [335]. CM promises to lessen the negative externalities of conventional agriculture without reducing meat qualities, which is helpful to consumers with environmental and/or ethical concerns [260]. This approach has the potential to drastically cut greenhouse gas emissions, land use, and water use in addition to doing away with the necessity for animal slaughter [10]. The existing concept of animal welfare may be impacted by the widespread usage of CM. Slaughter might be viewed as needless animal cruelty if it were possible to satisfy the demand for meat without killing animals. Making the avoidance of animal killing a high priority would mean abandoning even moderate conventional agricultural techniques or cultural activities that depart from this principle, such as hunting. The Pyrenees National Park in France/Spain, the Burren in Ireland, the Lake District National Park in the UK, and the Massif Central in France are just a few examples of cultural landscapes that have been developed through animal husbandry and are significant for regional identity and heritage [336,337].

While reducing the detrimental effects of conventional meat production, CM made under controlled settings utilizing cell culture technology has the ability to satisfy the world’s expanding protein needs [32,99,338]. Food safety can be enhanced and illness can be avoided by producing CM in a sterile manner. A severe problem linked with livestock husbandry is the predominance of bacteria such as *Escherichia coli*, *Salmonella*, and *Campylobacter* in meat and the spread of foodborne diseases of animal origin. Any indications of infection can be contained because CM is kept in a monitored and controlled environment [73].

Farm animals are regarded by scientists as sentient entities with both physical and psychological needs [339]. Stem cell collecting is another significant animal welfare issue. Muscle samples must be collected in order to create meat, which drastically lowers the number of animals killed [73]. Therefore, while CM does not totally emancipate animals, it effectively replaces traditional meat and mimics the eating experience without directly altering human cognitive processes [340]. The creation of specified media for the proliferation or differentiation of muscle cells has demonstrated encouraging outcomes recently and seems to be the recommended strategy. Finding natural, non-animal substitutes for fetal serum is a more straightforward, less costly, and promising approach [36]. However, when gathering tissue for a bone biopsy, ethical and animal welfare considerations are vital. Donor animals’ suffering and discomfort can be greatly reduced by using compassionate and less intrusive biopsy techniques. Animal welfare can be enhanced by the development of non-invasive or minimally invasive sample techniques, such as skin biopsies or fine needle aspiration biopsies [338].

One typical animal-derived component utilized as a fetal growth medium is bovine serum. With 200–400 proteins and many tiny compounds in varying amounts, it is thought to be a universal supplement. Its potential for contamination is unethical and unsustainable for CM [339]. Furthermore, bovine serum is expensive, and because it is derived from animals, its usage is contradictory with the suggested creation of animal-free [82]. Kolkmann et al.’s research [232] on modified serum for bovine myoblast culture has shown promise for use. In comparison to the gold standard culture medium, chemically defined media support 97% proliferation of primary bovine myoblast cells [341]. To prevent escalating already-existing disparities in food systems, it is essential to guarantee the accessibility and affordability of CM. Policies and subsidies may be necessary to ensure the provision of CM to all socioeconomic classes [336].

CM, like normal meat, does not violate the senses or emotions of animal lovers. The majority of rabbis believe meat raised in Judaism to be kosher; however, some contend that the cells must originate from an animal that was murdered in a kosher manner [305,342]. In Islam, CM is considered halal if no animal blood or serum is utilized during the production process and the cells are derived from an animal that was killed in accordance with halal regulations [305]. It is chosen for its safety, lack of infections, environmental friendliness, and ethics, ensuring the happiness of all food enthusiasts [32,73].

However, the manufacturing of CM may also have less evident implications on economic security, such as endangering the stability of the animal by-product supply, which could have detrimental repercussions on the market. According to Lee [343], producing synthetic alternatives to other animal products would be more environmentally damaging and less effective than traditional production. Conventional meat production may lose jobs as a result of the introduction of resource-efficient CM products to the market; naturally, this will mostly depend on market share and demand, as well as if farmers themselves can or will become CM producers (quality value: income security).

Ecological sustainability is compatible with some aspects of conventional agriculture and traditional meat production, which can contribute to enhancing resource efficiency, sustainability, and biodiversity. The problem of biodiversity is more complicated: while intensive agriculture is acknowledged to be a concern, certain agricultural systems and methods of producing meat can contribute to the preservation of biodiversity [336].

## 9. Commercial Production of CM

Many businesses, including Aleph Farms, Mosa Meat, Shiok Meats, Upside Foods, and others, are making significant expenditures in their CM research since it is a “hot topic” for investors, along with cultured seafood and poultry [344]. Institute (GFI) 2023 State of the Industry report, 10 new CM plants have been established in Asia, Australia, Europe, North America, and the Middle East [345,346]. Despite billions of dollars being invested and more than 150 businesses working on their solutions, many of these issues, particularly the techno-economic ones, remain unresolved [347]. The creation of an affordable, robust, food-grade, and animal-free cell culture medium is one of the biggest techno-economic difficulties [348]. Numerous elements, including social, psychological, and economic ones, can affect CM’s long-term commercial success. The longevity and profitability of this technology are significantly impacted by the numerous unresolved technological, social, economic, and other issues [85]. Along with the substantial funding needed for additional study in this area, production costs are a crucial concern [316,349].

Prior authorization based on a food safety risk assessment submitted to scientifically based, essentially independent food authorities or agencies is typically required for the market introduction of these so-called novel foods. Several nations, including Canada, Australia, the EU, Israel, and the UK [21,128,350], have developed regulations specifically governing the marketing of novel foods with the primary goal of ensuring a high standard of consumer health protection. Recalling the precautionary principle acknowledged in Article 7 of Regulation (EC) No. 178/2002 and the potential risks to consumer health as well as the livelihoods of the Italian agricultural sector, the government’s decision has sparked an intense political and scholarly discussion that also includes the potential future relationship between this national legislation and the previously mentioned EU Regulation on novel foods [128,351].

Singapore was the first country in the world to approve the commercial sale of meat produced from cell cultures in 2020. In 2024, the Singapore Food Agency (SFA) updated its regulations on novel foods to cover products without a documented history of safe use [57,352]. These regulations require producers planning to introduce such products to the market to submit a safety assessment to the agency, which serves as the basis for data analysis. For CM, the SFA assesses safety at three levels: the production process (cell lines, media, reagents, toxicology); the technological process and control systems (including contamination and hygiene compliance); and the final product, which must meet national food standards [57].

In 2022, the US FDA approved the commercialization of chicken CM, confirming its safety for consumers [353]. Companies present a general production flowchart, which begins with the creation of a cell bank, ensuring a uniform and controlled source of material for food production. The process involves cell multiplication and collection for further, conventional food processing. Companies also declare the use of a comprehensive food safety and quality system, including: (i) current good manufacturing practices (GMP), (ii) hazard analysis and risk-based control plan (HACCP), along with preventive and corrective actions for biological, chemical, and physical hazards, and (iii) in-process controls. According to the data provided, the systems and procedures implemented guarantee the safe production of CM. Documents indicate that no process step poses a risk of contamination that could adulterate the product, and the produced meat is as safe as its conventionally produced counterparts. The USDA-FSIS Food Safety and Inspection Service (FSIS) has developed guidelines for sampling for laboratory testing and product labeling.

In Canada, novel foods and food ingredients are considered novel under the Food and Drug Administration (FDA) regulations and require a pre-market safety assessment to demonstrate their safety before being placed on the market. Food produced using cellular agriculture methods will be produced similarly to the US, in accordance with general hygiene and safety principles, including GMP requirements and the HACCP system [354].

An EFSA document from 2024 states that, under EU law, food and ingredients produced using cell culture methods require authorization before being released for sale, and in many cases, an EFSA scientific opinion on their safety is also required. Due to the rapid development of technology, EFSA emphasizes the need to update risk assessment methods to effectively protect consumer health and their interests [355]. Despite progress at the EU level, in 2023 it presented a draft law banning food produced from animal cell or tissue cultures, including CM. The Italian government adopted a law in 2023 prohibiting the production, sale, import, distribution, use, and promotion of such “synthetic meat,” justifying concerns for consumer health and the protection of the domestic agricultural sector [356].

In Israel, commercialization of cultured beef has been permitted since 2024, subject to safety assessments, labeling requirements, and licensing. The Ministry announced an assessment of key aspects to support the development of the sector while protecting public health [357]. Australia and New Zealand, like Brazil, do not have specific detailed regulations on CM. General novel food regulations can be used to regulate this area [57].

Countries affiliated with the FAO and WHO have developed reports on cell-based food production. The 2024 report highlights six main categories of food safety issues as follows: (i) genetic stability of cells/cell lines; (ii) microbiological risk associated with cell lines; (iii) exposure to substances used in the production process; (iv) toxicity and allergenicity to the general population; (v) risk of post-harvest microbiological contamination; and (vi) chemical contamination/residue levels [358].

**Figure 3:**
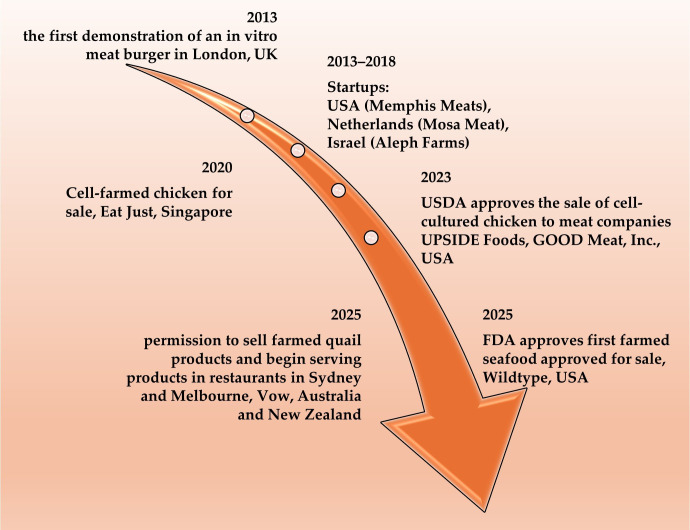
Calendar of development of the CM market [361,362,363,364].

The development of CM has advanced quickly over the last ten years (Figure 3). By the end of 2023, more than one hundred companies were engaged in CM technology research and development, developing ancillary services, and commercializing end products [316]. However, a number of factors, including nutritional quality, food safety, and sensory qualities, must be taken into account in addition to biologically matching typical meat [359]. Artificial intelligence (AI) has recently emerged as a crucial element of biomedical research [360]. Significant promise for cellular agriculture is suggested by its expanding involvement in organoid growth. The scalability and efficiency of culture could be greatly increased by combining AI with digital modeling to optimize cell culture media and tissue engineering for 3D meat production [193].

According to research, there is a sizable market for CM in many nations, and consumers in North America have a very favorable opinion of it [285,365]. Cultured beef is seen favorably in Italy, the fifth-largest meat producer in Europe [366]. Nonetheless, this product has raised more concerns in developing nations [365]. Three consumer segments have been established in China: Pioneers (32.4%), Accepters (41.9%), and Conservatives (25.7%). These groups differ significantly in terms of household size, age, income, and education [367,368].

Foods using cultured (lab-grown) muscle cells (CMC) have emerged as a result of pressure on conventional global food systems and social discourse on perceived drawbacks of animal production [2,369]. The CMC business claims to have improved animal welfare outcomes, had no effect on the environment, and drastically decreased the demand for natural resources [370,371,372]. The public understandably responds favorably to these statements. Significant technical difficulties in reproducing the nutrient-rich profile and sensory quality of natural meat are revealed by a detailed examination of the CMC business [18,373].

Chen et al. [25] claim that processes increasing scale in relation to meat bred in a farm they will be required large outlays financial in terms of costs capital related to equipment and objects, lines cellular and substrates breeding, contribution resources in raising qualifications, development knowledge and training, as well as development and dissemination of standards and management, and also increased resources such as water and energy.

Mosa Meat, a Dutch firm, was the first to introduce cultured beef to the general public. They produced cultured beef using stem cells from a cow that, after breeding and differentiating, became muscular strips. This business produced affordable CM by developing a medium free of bovine serum [285]. Memphis Meats, a California firm, created the first cultured meatballs using cultured beef in 2016. Their pilot facility has now started producing cultured beef and fowl [374,375].

JUST, a vegan food firm, has used cell cultures to produce clean chicken flesh. In 2019, JUST’s farmed chicken meat was priced at USD 50 per kilogram [374]. A food technology business called Memphis Meats successfully presented and manufactured CM products in 2016 [376]. They year 2018 saw the introduction of cell-cultured chicken flesh by Future Flesh Technologies, an Israeli business. This company lowered the cost of production per pound of chicken to USD 150 [170].

The main CM-producing companies are located in the following continents: 40% in Europe (Croatia, Czech Republic, Estonia, France, Germany, Israel, Italy, Netherlands, Russia, Spain, Switzerland, Turkey, England), 34% in North America (America and Canada), 15% in Asia (China, India, Japan, Singapore, South Korea), 6% in South America (Argentina, Brazil, Chile, Mexico), 3% in Oceania (Australia), and 2% in Africa (South Africa) [377].

Global capital investment in the industry has increased as the number of companies in the CM space has increased five-fold, from 12 to 60, between 2013 and 2020 [109]. According to the Good Food Institute [378] the number of companies increased to 156 by the end of 2022. The CM market size was estimated at USD 1.64 million in 2021 and is projected to reach USD 206.6 million and USD 2.79 billion by 2025 and 2030, respectively [379].

An accurate picture of the broader impacts of CMC foods is difficult to obtain because the industry is protected by proprietary licenses that do not allow for detailed life cycle assessment (LCA) [68,380,381]. In a study using ex-ante LCA, commercial-scale CMC food production in 2030 was modeled to be three times more efficient in producing natural meat substitutes compared to conventional animal production [70] myotube differentiation technologies [25,382]. Food scientists reduced production costs from USD 325,000 to USD 11.36 per 100 g serving of in vitro beef and USD 4.00 per 100 g of in vitro chicken breast [383,384]. Garrison et al., on the other hand, estimate the cost of 1 kg of farm-raised beef at USD 63/kg, based on the costs of a company producing 540 tons of meat annually, which would imply a retail price of USD 18 or more for a 0.14 kg hamburger [385]. The cost of production at this level could mean a price in a restaurant or supermarket above USD 100/kg [15]. Pasitka et al. estimate that it is possible to produce CM from chicken for USD 6.2/lb [386].

One of the most expensive aspects of CM production is the medium that provides essential nutrients for cell culture. Historically, animal serum-based media cost approximately EUR 175 per liter [387]. The French startup Gourmey and Deeplife’s collaboration to create a digital twin culture using AI is a model that enables thousands of virtual experiments, identifying optimal feed formulations and bioreactor conditions to maximize yield, minimize resource utilization, and enhance the sensory properties of CM. Reducing the cost of the culture medium to approximately 20 cents per liter allows for meat production at USD 3.43/lb [388]. The experience of animal meat companies can also be useful in reducing production costs. The startup Meatly, which is approved to sell cultivated chicken to animals in the UK, has reduced the cost of culture media to USD 0.30 per liter, which will be further reduced to just USD 0.02 on an industrial scale. Another startup producing meat for animals, BioCraft Pet Nutrition, has developed a plant-based growth medium that reduces the cost of its ingredients to USD 2–2.50 per pound.

In countries where CM is approved (Singapore, the USA, Israel, Australia), it appears in restaurants for promotional purposes and testing to gather feedback. In Singapore, chicken portions are sold for USD 23 per portion. Prices for CM dishes are typically comparable to premium restaurant dishes made with high-quality traditional meat. In most countries, CM is not yet available for regular sale. In the UK, only a product for dogs is available, and it is expected to be available for humans by 2027 [389].

### 9.1. Advantages of CM

Over the past ten years, CM—a conceptual product made from cultivated animal cells—has grown in favor because of the potential advantages it may have over traditional meat. Benefits are said to include improved animal protein production efficiency, the removal of animal welfare issues, and less of an influence on the environment through lower emissions and the usage of resources like land and water [236]. Theoretically, it is the best way to produce meat that is both environmentally sustainable and humane to animals, while still having the same flavor and nutritional value as conventional meat and offering extra advantages like controlled fat content and the absence of antibiotics and hormones used in the traditional meat industry [390]. CM is produced by cultivating animal cells in controlled settings, which reduces the need for traditional livestock farming and tackles important issues such resource inefficiency, animal welfare, environmental degradation, and the risk of foodborne illness [61,391,392].

The benefit of CM is that it may be made more biologically valuable by adding different vitamins, trace elements, amino acids, unsaturated fatty acids, etc. Additionally, CM cannot be a source of parasitic worms [147,393,394]. Genetic engineering has also been reported to help reduce alpha-gal meat allergy syndrome by removing the causative sugars from the cell surface [395].

Because CM is made under sterile conditions, it has a longer shelf life than traditional meat. CM’s sterility promotes its usage as a long-lasting, safe meat and lowers food waste [396]. However, CM processed in sterile circumstances can have a longer shelf life than normal meat while lowering transportation, cooling, and waste expenses, according to Gasteratos (2019) [397]. However, in order to produce meat products based on it, the CM production process includes not only the generation of cells and tissues but also the collection and purification of cells following manufacturing, storage, transportation, standardization, quality control, and food processing technologies [82]. The use of CM grown from animal cells eliminates animal slaughter, and additional benefits of CM include environmentally friendly factors: reduced greenhouse gas production, reduced land and water consumption for meat production. Research has been done in recent years to ascertain whether CM is generally accepted by consumers [317].

Because the slaughter procedure is skipped in the case of CM, the danger of contamination with pathogenic bacteria (such as *Salmonella*, *Campylobacter*, and *Escherichia coli*) is thought to be greatly decreased [73]. More research is needed to determine the source animal’s health status and the possibility of biological dangers such viruses and prions entering cell-cultured products [256]. The lack of unwanted smells, such as boar taint, which are brought on by the compounds androstenone and skat-ole present in the fat of male pigs reared traditionally, is a clear benefit of CM [16,18].

However, in recent years, these claims have faced increased criticism due to unfulfilled promises and lack of transparency [73,84,373,398].

### 9.2. Barriers to CM Production

For the proper development of the CM industry, the following issues are important: (1) expanding knowledge about CM and maximizing the development of technology; (2) improving product quality; (3) reducing production costs; (4) ensuring product safety; and (5) improving regulatory systems and ensuring good market access [256].

High production costs, complicated supply chain logistics, regulatory barriers, and issues with sensory qualities, nutritional quality, food safety, and customer acceptance are some of the main barriers to the industrialization of CM [229,377,399]. This large-scale CM production’s primary obstacle is its high cost. A viable CM bioprocess that yields a product that tastes and has the same nutritional value as current meat products is necessary for industrial-scale CM production to be feasible [390]. Tissue engineering has come a long way and will become a more significant part of the food business in the future. However, before it can be produced on a big scale and supplied at prices competitive with conventional meat, there are still technical and financial obstacles to overcome, such as scalability, cost, and regulatory approval. Additionally, before CM is generally accepted by consumers, there can be cultural barriers to overcome [36]. Although growing knowledge and awareness may encourage customers to buy engineered products, the largest disadvantage is the public’s fear of adopting genetic engineering techniques in cell culture systems to generate cell-based foods [400].

Perhaps even more importantly, CM production may only be feasible in countries with robust energy infrastructure and a highly educated workforce. CM could exacerbate economic inequality between and within countries. Skeptics fear that CM could exacerbate inequality between rich and poor, speculating that CM could cheaply feed the masses, leaving real meat in the hands of the wealthy [305]. The effects of switching from conventional meat to CM could vary for multiple stakeholders in the animal agriculture ecosystem, including farmers, consumers, rural residents, and tourism and hospitality businesses. Positive or negative impacts for each stakeholder could encompass economic, social, and environmental aspects [120,328,401,402]. According to Hocquette et al. (2025) [371], the future of CM is dubious when compared to alternative sustainable solutions like transitioning to plant-based, algae-based, or insect-based proteins. Concerns about CM range widely, from consumers’ general distaste for unfamiliar foods [261] to doubts about its safety and long-term effects [403,404]. Treich [14] expresses fear that meat grown in cultures breeding can “significantly influence on authorities” market [230]. He notes that the meat sector is already highly concentrated in the hands of a few actors, and that recent decades have seen a significant erosion of farmers’ control and autonomy in the food system [405,406]. New “alternative” innovations may create opportunities for new businesses, but the global protein sector may also become more concentrated [14] with specific companies (possibly from countries in the global North) [230] controlling the supply of CM products, as has been the case, for example, with genetically modified seeds. Because consumers are generally reluctant to change their dietary habits, a shift away from meat towards plant proteins is unlikely in the short term [407].

### 9.3. CM Production Safety

Cells derived from living animals used for CM production may carry viruses, bacteria, parasites, or prions that could potentially affect the health of the consumer [408,409]. At the stage of animal cell donation, it is necessary to control the quality of the animal feed (no meat and bone meal feeding), health history from creation, including breed, sex, age, biopsy site, data, medications administered, because biopsy protection performed by a qualified veterinarian is necessary before biopsy samples are taken [410]. Based on conventional meat production and aspects related to animal husbandry, *Brucella abortus*, *Mycobacterium bovis*, bovine spongiform encephalopathy (BSE) prion, and *Toxoplasma gondii* appear to pose the main threats at this stage. They appear to pose a threat to both conventional and CM [411]. Several limitations are associated with the use of genetic modification in CM. A notable issue concerns the possible stimulation of oncogenes and various mutations [412]. Contamination in cell culture can inhibit cell growth or introduce pathogens into CM, posing a health risk to consumers. Therefore, a more stringent sterile environment is required for CM production compared to conventional food processing environments. Various antibiotics are used to maintain this sterility by combating specific microbial contaminants. Commonly used antibiotics include penicillin and streptomycin, gentamicin, and antifungal agents, such as amphotericin B and plasmocin, are used to combat fungi and yeasts [413]. The use of antibiotics in meat production means that effective control measures must be implemented to reduce the possibility of their presence in the meat product [230,247,258,348,414,415,416,417].

On a large scale, the product will not be manufactured in a laboratory but at an industrial level, where it is impossible to completely eliminate potential hazards, especially those resulting from human error. This is a common problem with plant-based protein products. Indeed, as reported by Banach et al. [418], processing can introduce microbiological hazards such as *Staphylococcus aureus*, mainly through food handling (skin contact) or *Listeria monocytogenes* during processing because they can be found in the processing environment.

Introducing new food products carries the potential risk of triggering allergic reactions. Therefore, many countries require allergenicity assessments before new ingredients are allowed on the market to confirm that they do not pose a health risk to consumers [419]. CM contains the same key molecular components as conventional meat, meaning it can trigger allergic reactions in individuals who are allergic to certain meat species [305].The primary concern regarding food allergies stems from sensitization to specific carbohydrate structures, such as alpha galactose (alpha gal) [420]. For example, individuals with alpha gal syndrome—a rare allergy to a sugar molecule found in red meat [421] —as well as individuals allergic to poultry may experience adverse reactions after consuming cell-cultured products from the same species [422]. Symptoms of such allergies can range from mild skin reactions to severe anaphylaxis, emphasizing the need for clear, accurate, and reliable labeling of these products. The use of cultured cell lines may lead to the emergence of new proteins with previously unknown allergenic potential [423]. These proteins, resulting from genetic modifications or bypassing typical cellular aging mechanisms, may induce allergic reactions not identified in the original source tissue [424]. Another critical area may be the composition of the medium; even if allergenic proteins are removed during processing, there is a risk of trace amounts remaining. Using alternatives to serum hydrolysates composed of proteins with unrecognized allergenic potential may bind to the materials used to create the scaffolds. Many materials have been proposed for use in the production of CM, which also has allergenic properties. These include those that are often proposed as scaffold materials, such as wheat [200], soy protein and soy [16,170,425,426,427], albumin [428], peanuts [191,210,216,429], and chitosan [425,426]. The allergenicity of these materials requires control during meat cultivation and production [99], particularly with respect to how the processes themselves influence allergenicity. The safety of these natural materials is determined through thorough testing, which involves testing for potential allergens, toxins, and microbiological contaminants. Ensuring that these plant-derived scaffolds do not introduce any hazardous compounds into the cell culture or final product is essential to maintaining the integrity and safety of CBM [430]. Changes in bioreactor conditions may result in increased protein expression or the emergence of new protein forms. The next step in which allergenic substances may appear is related to improving the organoleptic properties and involves the addition of binders or color-enhancing substances.

The nutritional safety of CBM is a critical area of concern because its production processes involve novel techniques that can affect its nutritional composition. Unlike conventional meat, the micronutrient profile of CBM, particularly with respect to essential elements such as iron, vitamins, and fatty acids, remains an understudied area that requires further research [18].

There is a knowledge gap regarding the environmental impact as well as other potential risks, including the long-term effects of consuming CM on human health [373]. During the production process of CM, ingredients such as structural materials, nutrients, and modulators of cell function may be introduced, the adverse effects of which are not yet known. This is an aspect in common with plant proteins and (fungus-based) meat analogues [418,431]. In fact, as reported by Banach et al. (2023) [418] and Zhang et al. (2023) [431], an increased incidence of food allergies can occur in multiple ways: (I) when proteins are removed from their natural matrix and incorporated in higher amounts into other constructs; (II) by introducing proteins that are not normally consumed and cause primary sensitization or show cross-reactivity with immunoglobulins of existing allergens; (III) Induced sensitization to new proteins may lead to cross-reactivity events to foods that are currently not or rarely considered allergenic.

The choice of ingredients used must be critically assessed, taking into account their potential to introduce microbial contaminants into the bioprocess. Incidentally, the use of supports (microcarriers/scaffolds) made from plant protein should be assessed, considering that plant proteins can be a source of spore-forming bacteria [432]. Some different plant ingredients showed a high proportion of spores within their total aerobic mesophilic count [433]. Similarly, post-harvest CBM poses a risk of contamination during processing, packaging, and storage. Poor hygiene, improper handling, and contact with contaminated surfaces or equipment can introduce pathogens into the final product [434].

Given the numerous processes and inputs involved, it is essential to ensure that what remains in the final product intended for human consumption does not have undesirable consequences for human health. Therefore, each step of the CM production process must be monitored to identify potential sources of hazards and contaminants [435]. So far, the US Food and Drug Administration (FDA) [436] and the Singapore Food Agency of Singapore (SFA) [437] have published guidelines for assessing the safety of CM. However, these analyses are largely based on data provided by companies themselves, and regulatory guidelines on safety have been criticized for being vague and insufficiently defining the scope of the assessment. The reliability of the information is questionable, as companies often protect details of their production processes as trade secrets. Assessment of CM allergenicity is essential to prevent exposure of sensitive individuals to food allergens. In the case of foods without a history of safe use, there is a risk that the proteins contained in them may cross-react with known allergens or even induce the development of new forms of hypersensitivity [57,128]. Therefore, in CM production, it is essential to correctly identify hazards and implement a system based on the principles of Hazard Analysis and Critical Control Points (HACCP) to analyze and control biological, chemical, and physical risks at all stages, from raw material production to the final product [57,414]. Future CM packaging must maintain or even improve product quality. Furthermore, CM packaging must also take into account consumer preferences and expectations, such as convenience and attractive presentation [147].

## 10. Conclusions

CM production is in the early stages of technology development and is subject to high costs and uncertainty; public policy can accelerate progress by supporting research and innovation in culture media, bioreactors, renewable energy, and scale-up.

CM has gained popularity due to its potential benefits over conventional meat, including environmental benefits, including reduced water and land consumption, and lower greenhouse gas emissions. CM production also eliminates increasingly common concerns about animal welfare, irregularities in this area, and slaughter. Because the entire production process is planned down to the smallest detail, CM can be freely modified and even personalized to meet the needs of specific consumer groups. This allows for the addition of vitamins, amino acids, and so-called “healthy fats”, making it a functional food. Furthermore, the nature of production creates opportunities to reduce antibiotics, hormones, and the transmission of microbiological hazards and parasites. Sterile production extends shelf life and reduces the risk of bacteria such as *Salmonella* and *E. coli*.

The production process is complex and involves many stages (cultivation, purification, processing). This requires maintaining high standards of design, implementation, and control, developed infrastructure, the involvement of highly qualified personnel, and systems to ensure food safety, as concerns about biological threats (e.g., prions, viruses) persist. Eliminating barriers requires developing technology and knowledge about cell culture, reducing production costs, and streamlining regulations and market availability. Risks related to the concentration of technology in a limited number of companies and vulnerability to energy supply disruptions must be considered. Climate policy should recognize that cellular production is energy-intensive; its environmental impact will only be beneficial with a growing share of renewable energy sources. CM production requires clear, predictable regulations regarding food safety, quality standards, labeling, and oversight. A lack of consistent regulatory frameworks (EU, US, and Asia) can lead to trade barriers and hinder market development. High production costs and complex logistics remain a continuing problem, as CM design and production are largely experimental. Among the primary barriers to market entry and expansion are difficulties in changing consumer eating habits and issues with sensory and nutritional quality, which contribute to low consumer acceptance, especially with genetic engineering. Another factor limiting demand is insufficient consumer awareness. CM production also raises certain socio-economic concerns, as meat farming requires significant investment, creates the risk of deepening inequalities between countries and social classes, raises concerns about the influence on the market and the concentration of power in the hands of corporations, and the need to retrain some people employed in the production of slaughter animals. The development of CM may change the structure of demand for animal products, which will affect farmers’ incomes and local economies; therefore, mechanisms should be envisaged to mitigate the effects of the transformation, e.g., support for regenerative agriculture or new models of cooperation (e.g., farmers as suppliers of raw materials for breeding media).

## Figures and Tables

**Table 1:** Biomaterials used for CM scaffolds.

Type of Biomaterial	Source of Origin	Properties	Literature
Polysaccharides			
Alginate	brown algae	ability to generate hydrogels when exposed to divalent cations such as calcium; can serve as a supporting matrix that maintains a hydrated environment for cell development and differentiation	[149,150,151,152]
Fucoidan	brown algae	anti-inflammatory, antithrombotic and anticancer effects; biocompatibility and ability to form hydrogels biocompatibility and ability to form hydrogels	[153,154]
Starch	seeds and tubers of plants	thickening, gelling and stabilizing properties; a biocompatible, biodegradable and renewable material that supports cell growth and is suitable for ecological production	[151,155]
Carrageenan or carrageenans	red algae	Stabilizing properties, gelling agents and thickeners; highly effective in creating durable gels; Biocompatibility and ability to form different structures;	[152,156]
Agarose	red algae	forms a semi-solid gel at low concentrations, making it well suited for constructing scaffolds; Agarose gels are biocompatible and capable of maintaining a hydrated environment, which facilitates cell proliferation and differentiation.	[157,158,159]
Ulvan sulfated polysaccharide	green seaweed	immunomodulatory, antioxidant and antithrombotic properties	[160,161]
Cellulose	cell walls of land plants	can be processed to obtain a variety of derivatives, including hydrogels and scaffolds, which are used in tissue engineering and CM production; the permeability of the structures allows for the effective diffusion of oxygen and nutrients, thus facilitating cell development and proliferation	[151,162,163]
Pectin	cell walls of citrus peels and apple pomace	gelling agent, stabilizer and thickener; ability to form gels in the presence of calcium ions, pectin is a desirable material for building scaffolds in CM production	[164,165,166]
Guar gum	guar seeds (*Cyamopsis tetragonoloba*)	high water solubility and gelling properties; By forming stable gels, it supports cell growth in a hydrated environment; its biocompatibility and non-toxicity make it suitable for edible scaffolds, improving the structure and texture of the final product	[167,168,169]
Proteins			
Soy protein	soy	innate compatibility and biochemical similarity to the ECM; has excellent gelling and emulsifying properties; suitability for cell adhesion, proliferation and maturation;	[152,158,170,171]
Zein protein	corn	biocompatibility, flexibility and cellular compatibility; The ability to create strong, flexible films and fibers; to be used as a scaffold in the cultivation of meat with a meat-like consistency; Zein scaffolds support MSC adhesion, proliferation and differentiation	[172,173,174]
Cell-free plants			
	from decellularized plant tissues	natural abundance, biocompatibility and ability to create complex 3D structures; enabling the replication of structural and mechanical features of animal tissues	[175,176,177,178]
Biomaterials of animal origin			
Collagen	various connective tissues in animals	is considered the gold standard for scaffolds with properties similar to ECM; Thanks to its supporting structure, collagen provides an optimal environment for cell adhesion and proliferation;	[177,179,180,181]
Gelatine	produced by partial hydrolysis or heating of collagen	It is similar to collagen, yet easier to transform into other forms such as gels, films and sponges; supports the growth of various cell types and can form hydrogels that maintain a moist environment necessary for cellular activity	[151,158,181,182]
Hyaluronic acid (HAc)	extracellular matrix (ECM) of connective tissue	plays an important role in moisturizing tissues and facilitating cell communication; attracting and retaining water, thus creating a hydrated environment that promotes cell growth and differentiation;	[183,184]
Fibrin	fibrinogen	supports cell migration and tissue formation, highly biocompatible, promoting cell adhesion and growth; provides a scaffolding that resembles the body’s tissue structure, facilitating the development of well-organized and functional tissues	[185,186,187]
Chitosan	Chitin of crustacean shells	Biocompatibility, biodegradability and inherent antibacterial properties of chitosan; Provides structural rigidity, facilitates cell adhesion and proliferation, thus creating a favorable environment for tissue development;	[151,188,189]
Keratin	Animal hair and nails	strong biocompatibility and ability to support cell adhesion and proliferation, keratin creates an optimal environment for cell growth and differentiation	[29,140,190]

**Table 2:** Examples of activities aimed at modifying the quality of CM.

Nutritional Goal	Bioengineering Levers	Evidence Regarding Bioavailability and Safety	Literature
Nutrients and amino acid profile			
Maintaining a complete protein profile	Cell line selection; differentiation regulation; media optimization	Amino acid profile similar to conventional meat; no toxicological signals	[82]
Increasing the share of selected amino acids	Metabolic modifications; precursor supplementation	The possibility of modulating the AA profile has been confirmed in vitro; no clinical trials have been conducted.	[20,230]
Lipids and fatty acid profile			
Reduction in saturated fatty acids	Regulation of adipogenesis; use of vegetable oils	Possibility of modulating the lipid profile; need to assess oxidative stability	[170,231]
Enriched with omega-3 fatty acids	Algae oil addition; adipocyte engineering	Omega 3incorporation confirmed in in vitro models	[232,233]
Micronutrients (iron, B_12_, zinc)			
Providing heme iron	Co-cultures; recombinant heme	Bioavailability theoretically high; no in vivo studies	[234,235]
Providing vitamin B_12_	Media supplementation; immobilization B_12_	Supplementation required; stability unconfirmed	[18,77]
Optimizing zinc and selenium levels	Precise media supplementation	No risk signals; bioavailability dependent on chemical form	[42,236]
Texture, structure and digestibility			
Digestibility comparable to traditional meat	Optimization of the fiber and adipocyte ratio; control of maturation	In vitro digestion models indicate similar digestibility	[237,238]
Improved texture and sensory properties	Bioprinting; scaffolding with adjustable porosity	No data on the effect on bioavailability; safety unchanged	[170,239]
Microbiological and chemical safety			
zoonotic pathogens	Aseptic production; no contact with the animal	Microbiological risk significantly lower than in traditional meat	[231,235]
Elimination of antibiotic residues	Antibiotic-free production	No residue in commercial products	[230,233]
Chemical pollution control	Standardization of media and scaffolds	High purity; full toxicology evaluations required	[20,42]
Health-promoting functionality			
Enrichment with bioactive peptides	precursor protein expression	Theoretical data; no in vivo studies	[82,238]
Reduction in pro-inflammatory compounds	Control of cellular oxidative stress	Lower levels of lipid oxidation products in preliminary studies	[77,236]

## Data Availability

No new data were created or analyzed in this study. Data sharing is not applicable to this article.

## Abbreviations

The following abbreviations are used in this manuscript:

AI	artificial intelligence
BSE	bovine spongiform encephalopathy
CBM	cell-based meat
CM	cultured meat
CMC	cultured (lab-grown) muscle cells
CMT	cultured muscle tissue
ECM	extracellular matrix
FS	fat substitute
GMOs	genetically modified organisms
GMS	glycerol monostearate
HAc	hyaluronic acid
iPSCs	induced pluripotent stem cells
LCA	life cycle assessment
MSC	muscle satellite cells
NPF	natural plant fiber
PEG	polyethylene glycol
PGA	polygalacturonic acid
PUFA	polyunsaturated fatty acids
SFA	saturated fatty acids

## References

[1] Lee H.J., Yong H.I., Kim M., Choi Y.-S., Jo C. (2020). Status of Meat Alternatives and Their Potential Role in the Future Meat Market—A Review. Asian-Australas. J. Anim. Sci..

[2] Deliza R., Rodríguez B., Reinoso-Carvalho F., Lucchese-Cheung T. (2023). Cultured Meat: A Review on Accepting Challenges and Upcoming Possibilities. Curr. Opin. Food Sci..

[3] Hocquette J.-F. (2023). Consumer Perception of Livestock Production and Meat Consumption; an Overview of the Special Issue “Perspectives on Consumer Attitudes to Meat Consumption”. Meat Sci..

[4] Sanchez-Sabate R., Badilla-Briones Y., Sabaté J. (2019). Understanding Attitudes towards Reducing Meat Consumption for Environmental Reasons—A Qualitative Synthesis Review. Sustainability.

[5] Michel F., Hartmann C., Siegrist M. (2021). Consumers’ Associations, Perceptions and Acceptance of Meat and Plant-Based Meat Alternatives. Food Qual. Prefer..

[6] Smith K., Watson A.W., Lonnie M., Peeters W.M., Oonincx D., Tsoutsoura N., Simon-Miquel G., Szepe K., Cochetel N., Pearson A.G. (2024). Meeting the Global Protein Supply Requirements of a Growing and Ageing Population. Eur. J. Nutr..

[7] van Vliet S., Bain J.R., Muehlbauer M.J., Provenza F.D., Kronberg S.L., Pieper C.F., Huffman K.M. (2021). A Metabolomics Comparison of Plant-Based Meat and Grass-Fed Meat Indicates Large Nutritional Differences despite Comparable Nutrition Facts Panels. Sci. Rep..

[8] Cedeno F.R.P., Olubiyo O.J., Ferreira S. (2025). From Microbial Proteins to Cultivated Meat for Alternative Meat-like Products: A Review on Sustainable Fermentation Approaches. J. Biol. Eng..

[9] GFI (2021). State of the Industry Report. Cultivated Meat.

[10] Joo S.-T., Choi J.-S., Hur S.-J., Kim G.-D., Kim C.-J., Lee E.-Y., Bakhsh A., Hwang Y.-H. (2022). A Comparative Study on the Taste Characteristics of Satellite Cell Cultured Meat Derived from Chicken and Cattle Muscles. Food Sci. Anim. Resour..

[11] Li Y., Liu W., Li S., Zhang M., Yang F., Wang S. (2021). Porcine Skeletal Muscle Tissue Fabrication for Cultured Meat Production Using Three-Dimensional Bioprinting Technology. J. Future Foods.

[12] Ong S., Choudhury D., Naing M.W. (2020). Cell-Based Meat: Current Ambiguities with Nomenclature. Trends Food Sci. Technol..

[13] Manning L., Dooley J.J., Dunsford I., Goodman M.K., MacMillan T.C., Morgans L.C., Rose D.C., Sexton A.E. (2023). Threat or Opportunity? An Analysis of Perceptions of Cultured Meat in the UK Farming Sector. Front. Sustain. Food Syst..

[14] Treich N. (2021). Cultured Meat: Promises and Challenges. Environ. Resour. Econ..

[15] Peker A., İplikçioğlu Aral G., Orkan Ş., Aral Y. (2024). A Comprehensive Outlook on Cultured Meat and Conventional Meat Production. Ank. Üniversitesi Vet. Fakültesi Derg..

[16] Broucke K., Pamel E.V., Coillie E.V., Herman L., Royen G.V. (2023). Cultured Meat and Challenges Ahead: A Review on Nutritional, Technofunctional and Sensorial Properties, Safety and Legislation. Meat Sci..

[17] Thorrez L., Vandenburgh H. (2019). Challenges in the Quest for ‘Clean Meat’. Nat. Biotechnol..

[18] Fraeye I., Kratka M., Vandenburgh H., Thorrez L. (2020). Sensorial and Nutritional Aspects of Cultured Meat in Comparison to Traditional Meat: Much to Be Inferred. Front. Nutr..

[19] Kim M., Jung H.Y., Ellies-Oury M.-P., Chriki S., Hocquette J.-F., Jo C. (2024). Technological Aspects of Bridging the Gap Between Cell-Based Food and Conventional Meat. Meat Muscle Biol..

[20] Rubio N.R., Xiang N., Kaplan D.L. (2020). Plant-Based and Cell-Based Approaches to Meat Production. Nat. Commun..

[21] FAO (2023). Meat Market Review: Emerging Trends and Outlook.

[22] Kwasny T., Dobernig K., Riefler P. (2022). Towards Reduced Meat Consumption: A Systematic Literature Review of Intervention Effectiveness, 2001–2019. Appetite.

[23] Whitnall T., Pitts N. (2019). Global Trends in Meat Consumption. Agric. Commod..

[24] Quan L., Han H. (2025). Consumer Behavior toward Cultured Meat in the Foodservice Industry: Insights from IPA and fsQCA Analysis on Shifting Trends. J. Retail. Consum. Serv..

[25] Chen L., Guttieres D., Koenigsberg A., Barone P.W., Sinskey A.J., Springs S.L. (2022). Large-Scale Cultured Meat Production: Trends, Challenges and Promising Biomanufacturing Technologies. Biomaterials.

[26] Bjørndal T., Dey M., Tusvik A. (2024). Economic Analysis of the Contributions of Aquaculture to Future Food Security. Aquaculture.

[27] Costello C., Cao L., Gelcich S., Cisneros-Mata M.Á., Free C.M., Froehlich H.E., Golden C.D., Ishimura G., Maier J., Macadam-Somer I. (2020). The Future of Food from the Sea. Nature.

[28] Samy-Kamal M., Mehanna S.F. (2023). Evolution of Fishing Effort and Fishing Capacity during the Last Two Decades (2000–2019) in Egypt’s Marine Fisheries: Spotting the Fleet Overcapacity. Reg. Environ. Change.

[29] Kang H.S., Bang S., Lee H., Moon C.H., Gwon J.Y., Seo J.H., Cha G.D., Lee D.-H., Lee K.-Y., Hwang H. (2025). Development of Cultivated Fish Meat: Advances in Cellular Agriculture, Biomaterials, and Scaffolding Techniques. Trends Food Sci. Technol..

[30] Obirikorang K.A., Quagrainie K., Kassah J.E., Von Ahnen M. (2024). Editorial: Sustainable Aquaculture Production for Improved Food Security. Front. Sustain. Food Syst..

[31] Leroy F., Smith N.W., Adesogan A.T., Beal T., Iannotti L., Moughan P.J., Mann N. (2023). The Role of Meat in the Human Diet: Evolutionary Aspects and Nutritional Value. Anim. Front..

[32] Balasubramanian B., Liu W., Pushparaj K., Park S. (2021). The Epic of In Vitro Meat Production—A Fiction into Reality. Foods.

[33] Issara U., Park S., Park S. (2019). Determination of Fat Accumulation Reduction by Edible Fatty Acids and Natural Waxes In Vitro. Food Sci. Anim. Resour..

[34] Giglio F., Scieuzo C., Ouazri S., Pucciarelli V., Ianniciello D., Letcher S., Salvia R., Laginestra A., Kaplan D.L., Falabella P. (2024). A Glance into the Near Future: Cultivated Meat from Mammalian and Insect Cells. Small Sci..

[35] Stephens N., Ellis M. (2020). Cellular Agriculture in the UK: A Review [Version 1; Peer Review: 2 Approved, 2 Approved with Reservations]. Wellcome Open Res..

[36] Santos A.C.A., Camarena D.E.M., Roncoli Reigado G., Chambergo F.S., Nunes V.A., Trindade M.A., Stuchi Maria-Engler S. (2023). Tissue Engineering Challenges for Cultivated Meat to Meet the Real Demand of a Global Market. Int. J. Mol. Sci..

[37] Stubelj M., Gleščič E., Žvanut B., Širok K. (2025). Factors Influencing the Acceptance of Alternative Protein Sources. Appetite.

[38] Molotoks A., Smith P., Dawson T.P. (2021). Impacts of Land Use, Population, and Climate Change on Global Food Security. Food Energy Secur..

[39] Agostoni C., Baglioni M., La Vecchia A., Molari G., Berti C. (2023). Interlinkages between Climate Change and Food Systems: The Impact on Child Malnutrition—Narrative Review. Nutrients.

[40] Macdiarmid J.I., Whybrow S. (2019). Nutrition from a Climate Change Perspective. Proc. Nutr. Soc..

[41] Owino V., Kumwenda C., Ekesa B., Parker M.E., Ewoldt L., Roos N., Lee W.T., Tome D. (2022). The Impact of Climate Change on Food Systems, Diet Quality, Nutrition, and Health Outcomes: A Narrative Review. Front. Clim..

[42] Lynch J., Pierrehumbert R. (2019). Climate Impacts of Cultured Meat and Beef Cattle. Front. Sustain. Food Syst..

[43] Park H., Cho I., Heo S., Han K., Baek Y., Sim W., Jeong D.-W. (2025). Metabolomic Insights of Cultured Meat Compared to Conventional Meat. Sci. Rep..

[44] Food and Agriculture Organization of the United Nations (2025). Total Meat Production.

[45] Bourdrez V., Chriki S. (2022). Qualités Nutritionnelle, Organoleptique et Disposition à Payer Pour Les Alternatives à La Viande: Cas Des Analogues Végétaux, de La” Viande In Vitro” et Des Insectes. INRAE Prod. Anim..

[46] Romão B., Botelho R.B.A., Torres M.L., da Costa Maynard D., de Holanda M.E.M., Borges V.R.P., Raposo A., Zandonadi R.P. (2023). Nutritional Profile of Commercialized Plant-Based Meat: An Integrative Review with a Systematic Approach. Foods.

[47] Cutroneo S., Prandi B., Faccini A., Pellegrini N., Sforza S., Tedeschi T. (2023). Comparison of Protein Quality and Digestibility between Plant-Based and Meat-Based Burgers. Food Res. Int..

[48] Boukid F. (2021). Plant-Based Meat Analogues: From Niche to Mainstream. Eur. Food Res. Technol..

[49] Jahn S., Furchheim P., Strässner A.-M. (2021). Plant-Based Meat Alternatives: Motivational Adoption Barriers and Solutions. Sustainability.

[50] Siddiqui S.A., Bahmid N.A., Mahmud C.M.M., Boukid F., Lamri M., Gagaoua M. (2023). Consumer Acceptability of Plant-, Seaweed-, and Insect-Based Foods as Alternatives to Meat: A Critical Compilation of a Decade of Research. Crit. Rev. Food Sci. Nutr..

[51] Onwezen M.C., Bouwman E.P., Reinders M.J., Dagevos H. (2021). A Systematic Review on Consumer Acceptance of Alternative Proteins: Pulses, Algae, Insects, Plant-Based Meat Alternatives, and Cultured Meat. Appetite.

[52] Zhang C., Guan X., Yu S., Zhou J., Chen J. (2022). Production of Meat Alternatives Using Live Cells, Cultures and Plant Proteins. Curr. Opin. Food Sci..

[53] Leroy F., Barnard N.D. (2020). Children and Adults Should Avoid Consuming Animal Products to Reduce Risk for Chronic Disease: NO. Am. J. Clin. Nutr..

[54] Leroy F., Abraini F., Beal T., Dominguez-Salas P., Gregorini P., Manzano P., Rowntree J., van Vliet S. (2022). Animal Board Invited Review: Animal Source Foods in Healthy, Sustainable, and Ethical Diets—An Argument against Drastic Limitation of Livestock in the Food System. Animal.

[55] Asher K.E., Peters P. (2020). Meat Reduction, Vegetarianism, or Chicken Avoidance: US Omnivores’ Impressions of Three Meat-Restricted Diets. Br. Food J..

[56] Demartini E., Vecchiato D., Finos L., Mattavelli S., Gaviglio A. (2022). Would You Buy Vegan Meatballs? The Policy Issues around Vegan and Meat-Sounding Labelling of Plant-Based Meat Alternatives. Food Policy.

[57] Zandonadi R.P., Ramos M.C., Elias F.T.S., Guimarães N.S. (2025). Global Insights into Cultured Meat: Uncovering Production Processes, Potential Hazards, Regulatory Frameworks, and Key Challenges—A Scoping Review. Foods.

[58] Seah J.S.H., Singh S., Tan L.P., Choudhury D. (2022). Scaffolds for the Manufacture of Cultured Meat. Crit. Rev. Biotechnol..

[59] Aschemann-Witzel J., Ares G., Thøgersen J., Monteleone E. (2019). A Sense of Sustainability?—How Sensory Consumer Science Can Contribute to Sustainable Development of the Food Sector. Trends Food Sci. Technol..

[60] Stephens N., Sexton A.E., Driessen C. (2019). Making Sense of Making Meat: Key Moments in the First 20 Years of Tissue Engineering Muscle to Make Food. Front. Sustain. Food Syst..

[61] Srutee R., Sowmya R.S., Annapure U.S. (2022). Clean Meat: Techniques for Meat Production and Its Upcoming Challenges. Anim. Biotechnol..

[62] Liu J., Chriki S., Kombolo M., Santinello M., Pflanzer S.B., Hocquette É., Ellies-Oury M.-P., Hocquette J.-F. (2023). Consumer Perception of the Challenges Facing Livestock Production and Meat Consumption. Meat Sci..

[63] Bhat Z.F., Morton J.D., Mason S.L., Bekhit A.E.-D.A., Bhat H.F. (2019). Technological, Regulatory, and Ethical Aspects of In Vitro Meat: A Future Slaughter-Free Harvest. Compr. Rev. Food Sci. Food Saf..

[64] Gaydhane M.K., Mahanta U., Sharma C.S., Khandelwal M., Ramakrishna S. (2018). Cultured Meat: State of the Art and Future. Biomanufacturing Rev..

[65] Munteanu C., Mireşan V., Răducu C., Ihuţ A., Uiuiu P., Pop D., Neacşu A., Cenariu M., Groza I. (2021). Can Cultured Meat Be an Alternative to Farm Animal Production for a Sustainable and Healthier Lifestyle?. Front. Nutr..

[66] Tuomisto H.L., Teixeira de Mattos M.J. (2011). Environmental Impacts of Cultured Meat Production. Environ. Sci. Technol..

[67] Tuomisto H.L., Ryynänen T., Soccol C.R., Molento C.F.M., Reis G.G., Karp S.G. (2024). Environmental Impacts of Cultivated Meat. Cultivated Meat: Technologies, Commercialization and Challenges.

[68] Rodríguez Escobar M.I., Cadena E., Nhu T.T., Cooreman-Algoed M., De Smet S., Dewulf J. (2021). Analysis of the Cultured Meat Production System in Function of Its Environmental Footprint: Current Status, Gaps and Recommendations. Foods.

[69] Lin J.W.X., Maran N., Lim A.J., Ng S.B., Teo P.S. (2025). Current Challenges, and Potential Solutions to Increase Acceptance and Long-Term Consumption of Cultured Meat and Edible Insects—A Review. Future Foods.

[70] Sinke P., Swartz E., Sanctorum H., van der Giesen C., Odegard I. (2023). Ex-Ante Life Cycle Assessment of Commercial-Scale Cultivated Meat Production in 2030. Int. J. Life Cycle Assess..

[71] Sinke P., Odegard I. (2021). LCA of Cultivated Meat. Future Projections for Different Scenarios.

[72] Dueñas-Ocampo S., Eichhorst W., Newton P. (2023). Plant-Based and Cultivated Meat in the United States: A Review and Research Agenda through the Lens of Socio-Technical Transitions. J. Clean. Prod..

[73] Chriki S., Hocquette J.-F. (2020). The Myth of Cultured Meat: A Review. Front. Nutr..

[74] Gilbert W., Thomas L.F., Coyne L., Rushton J. (2021). Review: Mitigating the Risks Posed by Intensification in Livestock Production: The Examples of Antimicrobial Resistance and Zoonoses. Animal.

[75] Bernstein J., Dutkiewicz J. (2021). A Public Health Ethics Case for Mitigating Zoonotic Disease Risk in Food Production. Food Ethics.

[76] Arango L., Chaudhury S.H., Septianto F. (2023). The Role of Demand-Based Scarcity Appeals in Promoting Cultured Meat. Psychol. Mark..

[77] Santo R.E., Kim B.F., Goldman S.E., Dutkiewicz J., Biehl E.M.B., Bloem M.W., Neff R.A., Nachman K.E. (2020). Considering Plant-Based Meat Substitutes and Cell-Based Meats: A Public Health and Food Systems Perspective. Front. Sustain. Food Syst..

[78] Williams S.C.P. (2024). Customers Start Eating Lab-Grown Meat—With a Side of Uncertainty. Engineering.

[79] Kouarfaté B.B., Durif F.N. (2023). A Systematic Review of Determinants of Cultured Meat Adoption: Impacts and Guiding Insights. Br. Food J..

[80] Ozhava D., Bhatia M., Freman J., Mao Y. (2022). Sustainable Cell Sources for Cultivated Meat. J. ISSN.

[81] Zhang G., Zhao X., Li X., Du G., Zhou J., Chen J. (2020). Challenges and Possibilities for Bio-Manufacturing Cultured Meat. Trends Food Sci. Technol..

[82] Post M.J., Levenberg S., Kaplan D.L., Genovese N., Fu J., Bryant C.J., Negowetti N., Verzijden K., Moutsatsou P. (2020). Scientific, Sustainability and Regulatory Challenges of Cultured Meat. Nat. Food.

[83] Melzener L., Verzijden K.E., Buijs A.J., Post M.J., Flack J.E. (2021). Cultured Beef: From Small Biopsy to Substantial Quantity. J. Sci. Food Agric..

[84] Olenic M., Deelkens C., Heyman E., Vlieghere E.D., Zheng X., van Hengel J., Schauwer C.D., Devriendt B., Smet S.D., Thorrez L. (2025). Review: Livestock Cell Types with Myogenic Differentiation Potential: Considerations for the Development of Cultured Meat. Animal.

[85] Jairath G., Mal G., Gopinath D., Singh B. (2021). A Holistic Approach to Access the Viability of Cultured Meat: A Review. Trends Food Sci. Technol..

[86] (2021). Groundbreaking New Reports Reveal Massive Environmental Benefits, Cost-Competitiveness of Cultivated Meat. https://gfi-apac.org/groundbreaking-new-reports-reveal-massive-environmental-benefits-cost-competitiveness-of-cultivated-meat/.

[87] Choudhury D., Singh S., Seah J.S.H., Yeo D.C.L., Tan L.P. (2020). Commercialization of Plant-Based Meat Alternatives. Trends Plant Sci..

[88] Stout A.J., Mirliani A.B., Rittenberg M.L., Shub M., White E.C., Yuen J.S.K., Kaplan D.L. (2022). Simple and Effective Serum-Free Medium for Sustained Expansion of Bovine Satellite Cells for Cell Cultured Meat. Commun. Biol..

[89] Zidarič T., Milojević M., Vajda J., Vihar B., Maver U. (2020). Cultured Meat: Meat Industry Hand in Hand with Biomedical Production Methods. Food Eng. Rev..

[90] Sun A., Wu W., Soladoye O.P., Aluko R.E., Bak K.H., Fu Y., Zhang Y. (2022). Maillard Reaction of Food-Derived Peptides as a Potential Route to Generate Meat Flavor Compounds: A Review. Food Res. Int..

[91] Park S., Jung S., Heo J., Koh W.-G., Lee S., Hong J. (2021). Chitosan/Cellulose-Based Porous Nanofilm Delivering C-Phycocyanin: A Novel Platform for the Production of Cost-Effective Cultured Meat. ACS Appl. Mater. Interfaces.

[92] Park S., Jung S., Choi M., Lee M., Choi B., Koh W.-G., Lee S., Hong J. (2021). Gelatin MAGIC Powder as Nutrient-Delivering 3D Spacer for Growing Cell Sheets into Cost-Effective Cultured Meat. Biomaterials.

[93] Su L., Jing L., Zeng X., Chen T., Liu H., Kong Y., Wang X., Yang X., Fu C., Sun J. (2023). 3D-Printed Prolamin Scaffolds for Cell-Based Meat Culture. Adv. Mater..

[94] Dohmen R.G.J., Hubalek S., Melke J., Messmer T., Cantoni F., Mei A., Hueber R., Mitic R., Remmers D., Moutsatsou P. (2022). Muscle-Derived Fibro-Adipogenic Progenitor Cells for Production of Cultured Bovine Adipose Tissue. npj Sci. Food.

[95] Lee M., Park S., Choi B., Kim J., Choi W., Jeong I., Han D., Koh W.-G., Hong J. (2022). Tailoring a Gelatin/Agar Matrix for the Synergistic Effect with Cells to Produce High-Quality Cultured Meat. ACS Appl. Mater. Interfaces.

[96] Pasitka L., Cohen M., Ehrlich A., Gildor B., Reuveni E., Ayyash M., Wissotsky G., Herscovici A., Kaminker R., Niv A. (2023). Spontaneous Immortalization of Chicken Fibroblasts Generates Stable, High-Yield Cell Lines for Serum-Free Production of Cultured Meat. Nat. Food.

[97] Fish K.D., Rubio N.R., Stout A.J., Yuen J.S.K., Kaplan D.L. (2020). Prospects and Challenges for Cell-Cultured Fat as a Novel Food Ingredient. Trends Food Sci. Technol..

[98] Kim C.-J., Kim S.-H., Lee E.-Y., Son Y.-M., Bakhsh A., Hwang Y.-H., Joo S.-T. (2023). Optimal Temperature for Culturing Chicken Satellite Cells to Enhance Production Yield and Umami Intensity of Cultured Meat. Food Chem. Adv..

[99] Hadi J., Brightwell G. (2021). Safety of Alternative Proteins: Technological, Environmental and Regulatory Aspects of Cultured Meat, Plant-Based Meat, Insect Protein and Single-Cell Protein. Foods.

[100] Lee M., Park S., Choi B., Choi W., Lee H., Lee J.M., Lee S.T., Yoo K.H., Han D., Bang G. (2024). Cultured Meat with Enriched Organoleptic Properties by Regulating Cell Differentiation. Nat. Commun..

[101] Kuppusamy P., Kim D., Soundharrajan I., Hwang I., Choi K.C. (2021). Adipose and Muscle Cell Co-Culture System: A Novel In Vitro Tool to Mimic the In Vivo Cellular Environment. Biology.

[102] Pallaoro M., Modina S.C., Fiorati A., Altomare L., Mirra G., Scocco P., Di Giancamillo A. (2023). Towards a More Realistic In Vitro Meat: The Cross Talk between Adipose and Muscle Cells. Int. J. Mol. Sci..

[103] Wang Y., Zhuang D., Munawar N., Zan L., Zhu J. (2024). A Rich-Nutritious Cultured Meat via Bovine Myocytes and Adipocytes Co-Culture: Novel Prospect for Cultured Meat Production Techniques. Food Chem..

[104] Ma T., Ren R., Lv J., Yang R., Zheng X., Hu Y., Zhu G., Wang H. (2024). Transdifferentiation of Fibroblasts into Muscle Cells to Constitute Cultured Meat with Tunable Intramuscular Fat Deposition. eLife.

[105] Aimaletdinov A., Abyzova M., Kurilov I., Yuferova A., Rutland C., Rizvanov A., Zakirova E. (2022). Isolation, Culturing and 3D Bioprinting Equine Myoblasts. Biol. Commun..

[106] Kumar P., Sharma N., Narnoliya L.K., Verma A.K., Mehta N., Bhavsar P.P., Kumar A., Lee S.-J., Sazili A.Q. (2024). Recent Advances in In-Vitro Meat Production—A Review. Ann. Anim. Sci..

[107] Adi P., Mulyani R., Yudhistira B., Chang C.-K., Gavahian M., Hsieh C.-W. (2024). Designing Cultivated Meat: Overcoming Challenges in the Production Process and Developing Sustainable Packaging Solutions. Trends Food Sci. Technol..

[108] Marcus N., Klink-Lehmann J., Hartmann M. (2022). Exploring Factors Determining German Consumers’ Intention to Eat Meat Alternatives. Food Qual. Prefer..

[109] Guan X., Lei Q., Yan Q., Li X., Zhou J., Du G., Chen J. (2021). Trends and Ideas in Technology, Regulation and Public Acceptance of Cultured Meat. Future Foods.

[110] Fu W., Zhang H., Whaley J.E., Kim Y.-K. (2023). Do Consumers Perceive Cultivated Meat as a Sustainable Substitute to Conventional Meat? Assessing the Facilitators and Inhibitors of Cultivated Meat Acceptance. Sustainability.

[111] Chandrababu A., Puthumana J. (2024). CRISPR-Edited, Cell-Based Future-Proof Meat and Seafood to Enhance Global Food Security and Nutrition. Cytotechnology.

[112] Albrecht F.B., Ahlfeld T., Klatt A., Heine S., Gelinsky M., Kluger P.J. (2024). Biofabrication’s Contribution to the Evolution of Cultured Meat. Adv. Healthc. Mater..

[113] Guo X., Wang D., He B., Hu L., Jiang G. (2024). 3D Bioprinting of Cultured Meat: A Promising Avenue of Meat Production. Food Bioprocess Technol..

[114] Gurel M., Rathod N., Cabrera L.Y., Voyton S., Yeo M., Ozogul F., Ozbolat I.T. (2024). A Narrative Review: 3D Bioprinting of Cultured Muscle Meat and Seafood Products and Its Potential for the Food Industry. Trends Food Sci. Technol..

[115] Kang D.-H., Louis F., Liu H., Shimoda H., Nishiyama Y., Nozawa H., Kakitani M., Takagi D., Kasa D., Nagamori E. (2021). Engineered Whole Cut Meat-like Tissue by the Assembly of Cell Fibers Using Tendon-Gel Integrated Bioprinting. Nat. Commun..

[116] Wang Y., Wang S., Zhuang D., Zan L., Zhu J. (2025). Quercetin-Enriched Animal-Free Scaffolds for Promoted Cell Proliferation and Differentiation in Cultured Meat Production. Food Chem..

[117] Niu R., Xin Q., Lao J., Huang X., Chen Q., Yin J., Chen J., Liu D., Xu E. (2025). Topological Polymeric Glucosyl Nanoaggregates in Scaffold Enable High-Density Piscine Muscle Tissue. Biomaterials.

[118] Que C., Cai X., Xu Y., Yang J., Zhu L., Zhang L., Tang W., Zhu K., Li C. (2025). Advances of 3D/4D Printed Hydrogels for Sensory and Nutritional Enhancement of Cell-Cultured Meat. Food Res. Int..

[119] Qin Z., Li Z., Huang X., Du L., Li W., Gao P., Chen Z., Zhang J., Guo Z., Li Z. (2025). Advances in 3D and 4D Printing of Gel-Based Foods: Mechanisms, Applications, and Future Directions. Gels.

[120] Rasmussen M.K., Gold J., Kaiser M.W., Moritz J., Räty N., Rønning S.B., Ryynänen T., Skrivergaard S., Ström A., Therkildsen M. (2024). Critical Review of Cultivated Meat from a Nordic Perspective. Trends Food Sci. Technol..

[121] Lima T.L.S., da Costa G.F., do Alves R.N., de Araújo C.D.L., da Silva G.F.G., Ribeiro N.L., de Figueiredo C.F.V., de Andrade R.O. (2022). Vegetable Oils in Emulsified Meat Products: A New Strategy to Replace Animal Fat. Food Sci. Technol..

[122] Albergaria J.D.S., dos Santos A.E.A., Guadalupe J.L.M., de Araújo I.P., Copola A.G.L., Santos J.P.F., Jorge E.C., de Andrade L.O., da Silva A.B. (2025). Edible Microcapsules Containing Canola Oil for Cultivated Meat Production. Appl. Food Res..

[123] Zeng X., Meng Z., He J., Mao M., Li X., Chen P., Fan J., Li D. (2022). Embedded Bioprinting for Designer 3D Tissue Constructs with Complex Structural Organization. Acta Biomater..

[124] Berg J., Kurreck J. (2021). Clean Bioprinting—Fabrication of 3D Organ Models Devoid of Animal Components. Altex.

[125] Chotelersak K., Teerawongsuwan S., Suwan N., Saipin N., Jaisin Y., Suriyut J., Boonprom P., Dorn-in S., Rungsiwiwut R. (2024). In Vitro Cultured Meat: Nutritional Aspects for the Health and Safety of Future Foods. Trends Sci..

[126] Marques D.M.C., Jabouille M., Gusmão A., Leite M., Sanjuan-Alberte P., Ferreira F.C. (2025). Microalgae-Enriched (Bio)Inks for 3D Bioprinting of Cultured Seafood. npj Sci. Food.

[127] Schätzlein E., Blaeser A. (2022). Recent Trends in Bioartificial Muscle Engineering and Their Applications in Cultured Meat, Biorobotic Systems and Biohybrid Implants. Commun. Biol..

[128] Lanzoni D., Rebucci R., Formici G., Cheli F., Ragone G., Baldi A., Violini L., Sundaram T.S., Giromini C. (2024). Cultured Meat in the European Union: Legislative Context and Food Safety Issues. Curr. Res. Food Sci..

[129] Becerra M.O., Contreras L.M., Lo M.H., Díaz J.M., Herrera G.C. (2020). Lutein as a Functional Food Ingredient: Stability and Bioavailability. J. Funct. Foods.

[130] Kim D.-H., Wang Y., Jung H., Field R.L., Zhang X., Liu T.-C., Ma C., Fraser J.S., Brestoff J.R., Dyken S.J.V. (2023). A Type 2 Immune Circuit in the Stomach Controls Mammalian Adaptation to Dietary Chitin. Science.

[131] Papadimitriou N., Markozannes G., Kanellopoulou A., Critselis E., Alhardan S., Karafousia V., Kasimis J.C., Katsaraki C., Papadopoulou A., Zografou M. (2021). An Umbrella Review of the Evidence Associating Diet and Cancer Risk at 11 Anatomical Sites. Nat. Commun..

[132] Shikano A., Kuda T., Shibayama J., Toyama A., Ishida Y., Takahashi H., Kimura B. (2019). Effects of *Lactobacillus plantarum* Uruma-SU4 Fermented Green Loofah on Plasma Lipid Levels and Gut Microbiome of High-Fat Diet Fed Mice. Food Res. Int..

[133] Kobayashi K., Tanaka M., Tanabe S., Yatsukawa Y., Tanaka M., Suzuki T. (2018). Distinguishing Glutamic Acid in Foodstuffs and Monosodium Glutamate Used as Seasoning by Stable Carbon and Nitrogen Isotope Ratios. Heliyon.

[134] Fu Y., Liu J., Hansen E.T., Bredie W.L.P., Lametsch R. (2018). Structural Characteristics of Low Bitter and High Umami Protein Hydrolysates Prepared from Bovine Muscle and Porcine Plasma. Food Chem..

[135] Chi W., Cao L., Sun G., Meng F., Zhang C., Li J., Wang L. (2020). Developing a Highly pH-Sensitive ĸ-Carrageenan-Based Intelligent Film Incorporating Grape Skin Powder via a Cleaner Process. J. Clean. Prod..

[136] Yang J., Fan Y., Cui J., Yang L., Su H., Yang P., Pan J. (2021). Colorimetric Films Based on Pectin/Sodium Alginate/Xanthan Gum Incorporated with Raspberry Pomace Extract for Monitoring Protein-Rich Food Freshness. Int. J. Biol. Macromol..

[137] Khalil R.K.S., Abdelrahim D.S., Khattab S.A.N. (2024). Sustainable Utilization of Valorized Agro-Wastes for Active and Intelligent Packaging of Processed Meats. Food Hydrocoll..

[138] Levi S., Yen F.-C., Baruch L., Machluf M. (2022). Scaffolding Technologies for the Engineering of Cultured Meat: Towards a Safe, Sustainable, and Scalable Production. Trends Food Sci. Technol..

[139] Singh A., Kumar V., Singh S.K., Gupta J., Kumar M., Sarma D.K., Verma V. (2023). Recent Advances in Bioengineered Scaffold for In Vitro Meat Production. Cell Tissue Res..

[140] Wang Y., Zou L., Liu W., Chen X. (2023). An Overview of Recent Progress in Engineering Three-Dimensional Scaffolds for Cultured Meat Production. Foods.

[141] Kulus M., Jankowski M., Kranc W., Golkar Narenji A., Farzaneh M., Dzięgiel P., Zabel M., Antosik P., Bukowska D., Mozdziak P. (2023). Bioreactors, Scaffolds and Microcarriers and In Vitro Meat Production—Current Obstacles and Potential Solutions. Front. Nutr..

[142] Allan S.J., De Bank P.A., Ellis M.J. (2019). Bioprocess Design Considerations for Cultured Meat Production with a Focus on the Expansion Bioreactor. Front. Sustain. Food Syst..

[143] Tuomisto H.L., Allan S.J., Ellis M.J. (2022). Prospective Life Cycle Assessment of a Bioprocess Design for Cultured Meat Production in Hollow Fiber Bioreactors. Sci. Total Environ..

[144] Wang Y., Chen Y., Li Y., Zuo D., Huang X., Tian X., Li Y., Wang W. (2025). Collagen as a Diversely Structural Biomaterial: From Assembly Strategies to Potential Applications in Food Industry. Food Hydrocoll..

[145] Yin W., Sun Z., McClements D.J., Jin Z., Qiu C. (2025). Advances in 3D Scaffold Materials and Fabrication Techniques for Cultured Meat Production: A Review. Food Biosci..

[146] Ahmad K., Lim J.-H., Lee E.-J., Chun H.-J., Ali S., Ahmad S.S., Shaikh S., Choi I. (2021). Extracellular Matrix and the Production of Cultured Meat. Foods.

[147] Siddiqui S.A., Bahmid N.A., Karim I., Mehany T., Gvozdenko A.A., Blinov A.V., Nagdalian A.A., Arsyad M., Lorenzo J.M. (2022). Cultured Meat: Processing, Packaging, Shelf Life, and Consumer Acceptance. LWT.

[148] Lee S.Y., Lee D.Y., Yun S.H., Lee J., Mariano E., Park J., Choi Y., Han D., Kim J.S., Hur S.J. (2024). Current Technology and Industrialization Status of Cell-Cultivated Meat. J. Anim. Sci. Technol..

[149] Lee J., Lee H., Cheon K.-H., Park C., Jang T.-S., Kim H.-E., Jung H.-D. (2019). Fabrication of Poly(Lactic Acid)/Ti Composite Scaffolds with Enhanced Mechanical Properties and Biocompatibility via Fused Filament Fabrication (FFF)–Based 3D Printing. Addit. Manuf..

[150] Baheiraei N., Razavi M., Ghahremanzadeh R. (2023). Reduced Graphene Oxide Coated Alginate Scaffolds: Potential for Cardiac Patch Application. Biomater. Res..

[151] Zo S.M., Sood A., Won S.Y., Choi S.M., Han S.S. (2025). Structuring the Future of Cultured Meat: Hybrid Gel-Based Scaffolds for Edibility and Functionality. Gels.

[152] Xia P., Miyajima H., Fujita S. (2025). Development of Biomimetic Edible Scaffolds for Cultured Meat Based on the Traditional Freeze-Drying Method for Ito-Kanten (Japanese Freeze-Dried Agar). Gels.

[153] Oliveira C., Neves N.M., Reis R.L., Martins A., Silva T.H. (2020). A Review on Fucoidan Antitumor Strategies: From a Biological Active Agent to a Structural Component of Fucoidan-Based Systems. Carbohydr. Polym..

[154] Lee J.-H., Kim T.-K., Kang M.-C., Park M.-K., Park S.-H., Choi J.-S., Choi Y.-S. (2024). Effect of Crude Polysaccharides from Ecklonia Cava Hydrolysate on Cell Proliferation and Differentiation of Hanwoo Muscle Stem Cells for Cultured Meat Production. Foods.

[155] Prasopdee T., Sinthuvanich C., Chollakup R., Uttayarat P., Smitthipong W. (2021). The Albumin/Starch Scaffold and Its Biocompatibility with Living Cells. Mater. Today Commun..

[156] Dong Y., Wei Z., Xue C. (2021). Recent Advances in Carrageenan-Based Delivery Systems for Bioactive Ingredients: A Review. Trends Food Sci. Technol..

[157] Su T., Zhang M., Zeng Q., Pan W., Huang Y., Qian Y., Dong W., Qi X., Shen J. (2021). Mussel-Inspired Agarose Hydrogel Scaffolds for Skin Tissue Engineering. Bioact. Mater..

[158] Hong S.-J., Kim D.-H., Ryoo J.-H., Park S.-M., Kwon H.-C., Keum D.-H., Shin D.-M., Han S.-G. (2024). Influence of Gelatin on Adhesion, Proliferation, and Adipogenic Differentiation of Adipose Tissue-Derived Stem Cells Cultured on Soy Protein–Agarose Scaffolds. Foods.

[159] Wollschlaeger J.O., Maatz R., Albrecht F.B., Klatt A., Heine S., Blaeser A., Kluger P.J. (2022). Scaffolds for Cultured Meat on the Basis of Polysaccharide Hydrogels Enriched with Plant-Based Proteins. Gels.

[160] Shah S., Famta P., Shahrukh S., Jain N., Vambhurkar G., Srinivasarao D.A., Raghuvanshi R.S., Singh S.B., Srivastava S. (2023). Multifaceted Applications of Ulvan Polysaccharides: Insights on Biopharmaceutical Avenues. Int. J. Biol. Macromol..

[161] Yi X., Xie J., Mei J. (2025). Recent Advances in Marine-Derived Polysaccharide Hydrogels: Innovative Applications and Challenges in Emerging Food Fields. Polymers.

[162] Zennifer A., Senthilvelan P., Sethuraman S., Sundaramurthi D. (2021). Key Advances of Carboxymethyl Cellulose in Tissue Engineering & 3D Bioprinting Applications. Carbohydr. Polym..

[163] Narayanan K.B., Bhaskar R., Kim H., Han S.S. (2023). In Vitro Cytocompatibility Assessment of Novel 3D Chitin/Glucan- and Cellulose-Based Decellularized Scaffolds for Skin Tissue Engineering. Sustainability.

[164] Zhang J., Liu P., Wu A., Song Y., Li Q., Liao X., Zhao J. (2024). Towards Understanding Pectin-Protein Interaction and the Role of Pectin in Plant-Based Meat Analogs Constructing. LWT.

[165] Wu Y., Li Y., Yang Q., He C., Tang J., Shi L., Dai J., Zhang C. (2025). Lotus Fiber-Derived Scaffolds for Enhanced Cultured Meat Production: Quality and Sustainability. Bioact. Mater..

[166] Kim W.-J., Kim Y., Lu Y., Ovissipour R., Nitin N. (2025). Evaluation of Plant-Based Composite Materials as 3D Printed Scaffolds for Cell Growth and Proliferation in Cultivated Meat Applications. Food Hydrocoll..

[167] Verma D., Sharma S.K. (2021). Recent Advances in Guar Gum Based Drug Delivery Systems and Their Administrative Routes. Int. J. Biol. Macromol..

[168] Chiu K.-H., Li S.A., Schillberg S., Ngwa C.J. (2025). Plant-Based Alginate-Guar Gum-Konjac Glucomannan Scaffold with Enhanced Thermal Stability and Biocompatibility for Cultured Meat Production. Future Foods.

[169] Kim Y.H., Jeong Y., Chang Y.H. (2025). High Internal Phase Pickering Emulsion Gel Stabilized by Whey Protein Isolate/Rutin/Guar Gum Complex for 3D Bio-Inks. Food Res. Int..

[170] Ben-Arye T., Shandalov Y., Ben-Shaul S., Landau S., Zagury Y., Ianovici I., Lavon N., Levenberg S. (2020). Textured Soy Protein Scaffolds Enable the Generation of Three-Dimensional Bovine Skeletal Muscle Tissue for Cell-Based Meat. Nat. Food.

[171] Sui X., Zhang T., Jiang L. (2021). Soy Protein: Molecular Structure Revisited and Recent Advances in Processing Technologies. Annu. Rev. Food Sci. Technol..

[172] Glusac J., Fishman A. (2021). Enzymatic and Chemical Modification of Zein for Food Application. Trends Food Sci. Technol..

[173] Huang X., Zeng J., Wang Y. (2023). Comparison of the Enhanced Attachment and Proliferation of the Human Mesenchymal Stem Cells on the Biomimetic Nanopatterned Surfaces of Zein, Silk Fibroin, and Gelatin. J. Biomed. Mater. Res. B Appl. Biomater..

[174] Melzener L., Spaans S., Hauck N., Pötgens A.J.G., Flack J.E., Post M.J., Doğan A. (2023). Short-Stranded Zein Fibers for Muscle Tissue Engineering in Alginate-Based Composite Hydrogels. Gels.

[175] Jones J.D., Rebello A.S., Gaudette G.R. (2021). Decellularized Spinach: An Edible Scaffold for Laboratory-Grown Meat. Food Biosci..

[176] Arslan Y., Paradiso A., Celiktas N., Erdogan T., Yesil-Celiktas O., Swieszkowski W. (2023). Bioinspired Microstructures through Decellularization of Plants for Tissue Engineering Applications. Eur. Polym. J..

[177] Bektas C., Lee K., Jackson A., Bhatia M., Mao Y. (2024). Bovine Placentome-Derived Extracellular Matrix: A Sustainable 3D Scaffold for Cultivated Meat. Bioengineering.

[178] Lu H., Ying K., Shi Y., Liu D., Chen Q. (2022). Bioprocessing by Decellularized Scaffold Biomaterials in Cultured Meat: A Review. Bioengineering.

[179] Koo Y.W., Lim C.S., Darai A., Lee J., Kim W., Han I., Kim G.H. (2023). Shape-Memory Collagen Scaffold Combined with Hyaluronic Acid for Repairing Intervertebral Disc. Biomater. Res..

[180] Jaime-Rodríguez M., Del Prado-Audelo M.L., Sosa-Hernández N.A., Anaya-Trejo D.P., Villarreal-Gómez L.J., Cabrera-Ramírez Á.H., Ruiz-Aguirre J.A., Núñez-Tapia I., Puskar M., Marques dos Reis E. (2025). Evaluation of Biocompatible Materials for Enhanced Mesenchymal Stem Cell Expansion: Collagen-Coated Alginate Microcarriers and PLGA Nanofibers. Biomolecules.

[181] Han J.H., Jang S.W., Kim Y.R., Na G.R., Park J.H., Choi H.W. (2024). Comparative Analysis of Different Extracellular Matrices for the Maintenance of Bovine Satellite Cells. Animals.

[182] Schneider K.H., Goldberg B.J., Hasturk O., Mu X., Dötzlhofer M., Eder G., Theodossiou S., Pichelkastner L., Riess P., Rohringer S. (2023). Silk Fibroin, Gelatin, and Human Placenta Extracellular Matrix-Based Composite Hydrogels for 3D Bioprinting and Soft Tissue Engineering. Biomater. Res..

[183] Wang Y., Wang J., Ma M., Gao R., Wu Y., Zhang C., Huang P., Wang W., Feng Z., Gao J. (2024). Hyaluronic-Acid-Nanomedicine Hydrogel for Enhanced Treatment of Rheumatoid Arthritis by Mediating Macrophage–Synovial Fibroblast Cross-Talk. Biomater. Res..

[184] Lamparelli E.P., Casagranda V., Pressato D., Maffulli N., Della Porta G., Bellini D. (2022). Synthesis and Characterization of a Novel Composite Scaffold Based on Hyaluronic Acid and Equine Type I Collagen. Pharmaceutics.

[185] Tang X., Deng G., Yang L., Wang X., Xiang W., Zou Y., Lu N. (2024). Konjac Glucomannan-Fibrin Composite Hydrogel as a Model for Ideal Scaffolds for Cell-Culture Meat. Food Res. Int..

[186] Geurs I., Vlieghere E.D., Grootaert C., Tzompa-Sosa D.A., Philips C., Bray F., Rolando C., Smet S.D., Dewettinck K., Vlierberghe S.V. (2025). The Extracellular Matrix of Beef: A Characterization towards a 3D Scaffold for Cultured Meat. Food Struct..

[187] Alam A.M.M.N., Kim C.-J., Kim S.-H., Kumari S., Lee E.-Y., Hwang Y.-H., Joo S.-T. (2024). Scaffolding Fundamentals and Recent Advances in Sustainable Scaffolding Techniques for Cultured Meat Development. Food Res. Int..

[188] Wang C., Makvandi P., Zare E.N., Tay F.R., Niu L. (2020). Advances in Antimicrobial Organic and Inorganic Nanocompounds in Biomedicine. Adv. Ther..

[189] Wang Y., Zhong Z., Munawar N., Wang R., Zan L., Zhu J. (2024). Production of Green-Natural and “Authentic” Cultured Meat Based on Proanthocyanidins-Dialdehyde Chitosan-Collagen Ternary Hybrid Edible Scaffolds. Food Res. Int..

[190] Li L., Zhang Y., Xu Z., Yan S., Yan N., Zhao C., Niu Y., Ding S., Zhou G., Chen L. (2025). Engineering Scaffold-Cell Interactions for Cultured Meat: Mechanisms, Materials, and Emerging AI-Driven Strategies. Trends Food Sci. Technol..

[191] Zheng Y.-Y., Chen Y., Zhu H.-Z., Li C.-B., Song W.-J., Ding S.-J., Zhou G.-H. (2022). Production of Cultured Meat by Culturing Porcine Smooth Muscle Cells In Vitro with Food Grade Peanut Wire-Drawing Protein Scaffold. Food Res. Int..

[192] Bomkamp C., Skaalure S.C., Fernando G.F., Ben-Arye T., Swartz E.W., Specht E.A. (2022). Scaffolding Biomaterials for 3D Cultivated Meat: Prospects and Challenges. Adv. Sci..

[193] Kumar A., Sood A., Han S.S. (2023). Technological and Structural Aspects of Scaffold Manufacturing for Cultured Meat: Recent Advances, Challenges, and Opportunities. Crit. Rev. Food Sci. Nutr..

[194] Ianovici I., Zagury Y., Redenski I., Lavon N., Levenberg S. (2022). 3D-Printable Plant Protein-Enriched Scaffolds for Cultivated Meat Development. Biomaterials.

[195] Su L., Jing L., Zeng S., Fu C., Huang D. (2024). Sorghum Prolamin Scaffolds-Based Hybrid Cultured Meat with Enriched Sensory Properties. J. Agric. Food Chem..

[196] Murugan P., Yap W.S., Ezhilarasu H., Suntornnond R., Le Q.B., Singh S., Seah J.S.H., Tan P.L., Zhou W., Tan L.P. (2024). Decellularised Plant Scaffolds Facilitate Porcine Skeletal Muscle Tissue Engineering for Cultivated Meat Biomanufacturing. npj Sci. Food.

[197] Hong T.K., Do J.T. (2024). Generation of Chicken Contractile Skeletal Muscle Structure Using Decellularized Plant Scaffolds. ACS Biomater. Sci. Eng..

[198] Chen Z., Xiong W., Guo Y., Jin X., Wang L., Ge C., Tan W., Zhou Y. (2024). Three-Dimensional Pore Structure of the Decellularized Parsley Scaffold Regulates Myogenic Differentiation for Cell Cultured Meat. J. Food Sci..

[199] Chen X., Li L., Chen L., Shao W., Chen Y., Fan X., Liu Y., Tang C., Ding S., Xu X. (2023). Tea Polyphenols Coated Sodium Alginate-Gelatin 3D Edible Scaffold for Cultured Meat. Food Res. Int..

[200] Xiang N., Yuen J.S.K., Stout A.J., Rubio N.R., Chen Y., Kaplan D.L. (2022). 3D Porous Scaffolds from Wheat Glutenin for Cultured Meat Applications. Biomaterials.

[201] Chen Y., Zhang W., Ding X., Ding S., Tang C., Zeng X., Wang J., Zhou G. (2024). Programmable Scaffolds with Aligned Porous Structures for Cell Cultured Meat. Food Chem..

[202] Lee S.-H., Choi J. (2024). The Need for Research on the Comparison of Sensory Characteristics between Cultured Meat Produced Using Scaffolds and Meat. Food Sci. Anim. Resour..

[203] Sun Z., Yin W., McClements D.J., Chen B., Ji H., Jin Z., Qiu C. (2025). Insight into Cultured Meat: The Effect of Anisotropy and the Use of Polysaccharides and Proteins from Non-Animal Origins for Scaffold Design. Carbohydr. Polym..

[204] Farshidfar N., Iravani S., Varma R.S. (2023). Alginate-Based Biomaterials in Tissue Engineering and Regenerative Medicine. Mar. Drugs.

[205] Reig-Vano B., Tylkowski B., Montané X., Giamberini M. (2021). Alginate-Based Hydrogels for Cancer Therapy and Research. Int. J. Biol. Macromol..

[206] Bomkamp C., Musgrove L., Marques D.M.C., Fernando G.F., Ferreira F.C., Specht E.A. (2023). Differentiation and Maturation of Muscle and Fat Cells in Cultivated Seafood: Lessons from Developmental Biology. Mar. Biotechnol..

[207] Moeini A., Pedram P., Fattahi E., Cerruti P., Santagata G. (2022). Edible Polymers and Secondary Bioactive Compounds for Food Packaging Applications: Antimicrobial, Mechanical, and Gas Barrier Properties. Polymers.

[208] Wei Z., Dai S., Huang J., Hu X., Ge C., Zhang X., Yang K., Shao P., Sun P., Xiang N. (2023). Soy Protein Amyloid Fibril Scaffold for Cultivated Meat Application. ACS Appl. Mater. Interfaces.

[209] Xiang N., Yao Y., Yuen J.S.K., Stout A.J., Fennelly C., Sylvia R., Schnitzler A., Wong S., Kaplan D.L. (2022). Edible Films for Cultivated Meat Production. Biomaterials.

[210] Song W.-J., Liu P.-P., Zheng Y.-Y., Meng Z.-Q., Zhu H.-Z., Tang C.-B., Li H.-X., Ding S.-J., Zhou G.-H. (2022). Production of Cultured Fat with Peanut Wire-Drawing Protein Scaffold and Quality Evaluation Based on Texture and Volatile Compounds Analysis. Food Res. Int..

[211] Liu S., Hua S., Gu X., Cai P., Zhou Y., Wang Y., Zhou M., Shan T. (2024). Production of Sodium Alginate-Gelatin Composite Hydrogel-Based 3D Cultured Fat with Low Cholesterol and High Polyunsaturated Fatty Acids. Food Hydrocoll..

[212] Carson C., Macias-Velasco J.F., Gunawardana S., Miranda M.A., Oyama S., Pierre C.L.S., Schmidt H., Wayhart J.P., Lawson H.A. (2020). Brown Adipose Expansion and Remission of Glycemic Dysfunction in Obese SM/J Mice. Cell Rep..

[213] Xu Z., You W., Zhou Y., Chen W., Wang Y., Shan T. (2019). Cold-Induced Lipid Dynamics and Transcriptional Programs in White Adipose Tissue. BMC Biol..

[214] Louis F., Furuhashi M., Yoshinuma H., Takeuchi S., Matsusaki M. (2023). Mimicking Wagyu Beef Fat in Cultured Meat: Progress in Edible Bovine Adipose Tissue Production with Controllable Fatty Acid Composition. Mater. Today Bio.

[215] Gu X., Hua S., Liu S., Zhou Y., Tan L.P., Wang Y., Zhou M., Shan T. (2026). A Konjac Glucomannan/Alginate-Based Hydrogel for Cultured Fat Production with Enhanced Lipo-Nutritional Features. Food Hydrocoll..

[216] Zheng Y.-Y., Shi Y.-F., Zhu H.-Z., Ding S.-J., Zhou G.-H. (2022). Quality Evaluation of Cultured Meat with Plant Protein Scaffold. Food Res. Int..

[217] Iravani S., Varma R.S. (2019). Plants and Plant-Based Polymers as Scaffolds for Tissue Engineering. Green Chem..

[218] Jahangirian H., Azizi S., Rafiee-Moghaddam R., Baratvand B., Webster T.J. (2019). Status of Plant Protein-Based Green Scaffolds for Regenerative Medicine Applications. Biomolecules.

[219] Levi S., Zernov A., Martin P., Baruch L., Zussman E., Machluf M. (2026). Not Just a Protein Source: Chickpea Protein-Based Scaffolds for Cultured Meat. Food Hydrocoll..

[220] Kolodkin-Gal I., Dash O., Rak R. (2024). Probiotic Cultivated Meat: Bacterial-Based Scaffolds and Products to Improve Cultivated Meat. Trends Biotechnol..

[221] Allan S.J., Ellis M.J., De Bank P.A. (2021). Decellularized Grass as a Sustainable Scaffold for Skeletal Muscle Tissue Engineering. J. Biomed. Mater. Res. A.

[222] Thyden R., Perreault L.R., Jones J.D., Notman H., Varieur B.M., Patmanidis A.A., Dominko T., Gaudette G.R. (2022). An Edible, Decellularized Plant Derived Cell Carrier for Lab Grown Meat. Appl. Sci..

[223] Harris A.F., Lacombe J., Zenhausern F. (2021). The Emerging Role of Decellularized Plant-Based Scaffolds as a New Biomaterial. Int. J. Mol. Sci..

[224] Kim D., Kim M., Lee C., Jang H., Kim W., Park J.H. (2025). Decellularized Extracellular Matrix Scaffolds from *Pleurotus ferulae* Mushrooms for Sustainable Production of Steak-like Cultured Meat with Authentic Texture. Mater. Today Bio.

[225] Liu Y., Zhou Y., Jia D., Yang Z., Li D., Li W., Liu B. (2025). Insight into the Aluminum Dopant-Induced Structure and Mechanical Property Variation in Amorphous SiBCN Ceramics. J. Mater. Sci. Technol..

[226] Singh A., Singh S.K., Kumar V., Gupta J., Kumar M., Sarma D.K., Singh S., Kumawat M., Verma V. (2024). Derivation and Characterization of Novel Cytocompatible Decellularized Tissue Scaffold for Myoblast Growth and Differentiation. Cells.

[227] Liu Y., Gao A., Wang T., Zhang Y., Zhu G., Ling S., Wu Z., Jin Y., Chen H., Lai Y. (2025). Growing Meat on Autoclaved Vegetables with Biomimetic Stiffness and Micro-Patterns. Nat. Commun..

[228] Choi K.-H., Yoon J.W., Kim M., Lee H.J., Jeong J., Ryu M., Jo C., Lee C.-K. (2021). Muscle Stem Cell Isolation and In Vitro Culture for Meat Production: A Methodological Review. Compr. Rev. Food Sci. Food Saf..

[229] Xie Y., Cai L., Ding S., Wang C., Wang J., Ibeogu I.H., Li C., Zhou G. (2025). An Overview of Recent Progress in Cultured Meat: Focusing on Technology, Quality Properties, Safety, Industrialization, and Public Acceptance. J. Nutr..

[230] Stephens N., Silvio L.D., Dunsford I., Ellis M., Glencross A., Sexton A. (2018). Bringing Cultured Meat to Market: Technical, Socio-Political, and Regulatory Challenges in Cellular Agriculture. Trends Food Sci. Technol..

[231] World Health Organization (2023). Food Safety Aspects of Cell-Based Food.

[232] Kolkmann A.M., Post M.J., Rutjens M.A.M., van Essen A.L.M., Moutsatsou P. (2020). Serum-Free Media for the Growth of Primary Bovine Myoblasts. Cytotechnology.

[233] Frezal C., Nenert C., Gay H. (2022). Meat Protein Alternatives: Opportunities and Challenges for Food Systems’ Transformation. OECD Food, Agriculture and Fisheries Papers.

[234] Bryant C., Barnett J. (2018). Consumer Acceptance of Cultured Meat: A Systematic Review. Meat Sci..

[235] Turck D., Bresson J.-L., Burlingame B., Dean T., Fairweather-Tait S., Heinonen M., Hirsch-Ernst K.I., EFSA Panel on Dietetic Products, Nutrition and Allergies (NDA) Products, Nutrition, Allergies (NDA) (2021). Guidance on the Preparation and Submission of an Application for Authorisation of a Novel Food in the Context of Regulation (EU) 2015/2283 (Revision 1)2. EFSA J..

[236] Tuomisto H.L. (2019). The Eco-friendly Burger: Could Cultured Meat Improve the Environmental Sustainability of Meat Products?. EMBO Rep..

[237] Dekkers B.L., Boom R.M., van der Goot A.J. (2018). Structuring Processes for Meat Analogues. Trends Food Sci. Technol..

[238] Zhang P., Zhao X., Zhang S., Li G., Midgley A.C., Fang Y., Zhao M., Nishinari K., Yao X. (2024). The Important Role of Cellular Mechanical Microenvironment in Engineering Structured Cultivated Meat: Recent Advances. Curr. Res. Food Sci..

[239] O’Neill E.N., Cosenza Z.A., Baar K., Block D.E. (2021). Considerations for the Development of Cost-Effective Cell Culture Media for Cultivated Meat Production. Compr. Rev. Food Sci. Food Saf..

[240] Clinquart A., Ellies-Oury M.P., Hocquette J.F., Guillier L., Santé-Lhoutellier V., Prache S. (2022). Review: On-Farm and Processing Factors Affecting Bovine Carcass and Meat Quality. Animal.

[241] Stout A.J., Mirliani A.B., Soule-Albridge E.L., Cohen J.M., Kaplan D.L. (2020). Engineering Carotenoid Production in Mammalian Cells for Nutritionally Enhanced Cell-Cultured Foods. Metab. Eng..

[242] Yen F.-C., Glusac J., Levi S., Zernov A., Baruch L., Davidovich-Pinhas M., Fishman A., Machluf M. (2023). Cultured Meat Platform Developed through the Structuring of Edible Microcarrier-Derived Microtissues with Oleogel-Based Fat Substitute. Nat. Commun..

[243] Martínez L., Ros G., Nieto G. (2018). Fe, Zn and Se Bioavailability in Chicken Meat Emulsions Enriched with Minerals, Hydroxytyrosol and Extra Virgin Olive Oil as Measured by Caco-2 Cell Model. Nutrients.

[244] Li X., Zhang G., Zhao X., Zhou J., Du G., Chen J. (2020). A Conceptual Air-Lift Reactor Design for Large Scale Animal Cell Cultivation in the Context of In Vitro Meat Production. Chem. Eng. Sci..

[245] Melios S., Grasso S., Bolton D., Crofton E. (2024). A Comparison of the Sensory Characteristics of Plant-Based, Nitrite-Free, Dry-Cured and Brine-Cured Bacon Rashers with Temporal Dominance of Sensations and Partial Napping with Ultra-Flash Profiling. LWT.

[246] Melios S., Grasso S., Bolton D., Crofton E. (2024). Sensory Characterisation of Meatless and Nitrite-Free Cooked Ham Alternatives in Comparison to Conventional Counterparts: Temporal Dominance of Sensations and Partial Napping with Ultra-Flash Profiling. Food Res. Int..

[247] Lee S.Y., Kang H.J., Lee D.Y., Kang J.H., Ramani S., Park S., Hur S.J. (2021). Principal Protocols for the Processing of Cultured Meat. J. Anim. Sci. Technol..

[248] Paredes J., Cortizo-Lacalle D., Imaz A.M., Aldazabal J., Vila M. (2022). Application of Texture Analysis Methods for the Characterization of Cultured Meat. Sci. Rep..

[249] Post M.J., Hocquette J.-F. (2017). New Sources of Animal Proteins: Cultured Meat. New Aspects of Meat Quality.

[250] Mateti T., Laha A., Shenoy P. (2022). Artificial Meat Industry: Production Methodology, Challenges, and Future. JOM.

[251] Wang Y., Yang X., Li L. (2024). Formation of pH-Responsive Hydrogel Beads and Their Gel Properties: Soybean Protein Nanofibers and Sodium Alginate. Carbohydr. Polym..

[252] Singh A., Verma V., Kumar M., Kumar A., Sarma D.K., Singh B., Jha R. (2022). Stem Cells-Derived In Vitro Meat: From Petri Dish to Dinner Plate. Crit. Rev. Food Sci. Nutr..

[253] Roy B.C., Bruce H.L. (2024). Contribution of Intramuscular Connective Tissue and Its Structural Components on Meat Tenderness-Revisited: A Review. Crit. Rev. Food Sci. Nutr..

[254] Wu D., Pang S., Bäther S., Woelken L., Abyzova M., Morales-Dalmau J., Haibel A., Jia Y., Berg J., Kaufer B.B. (2026). Embedded Bioprinting Enables Precise Fabrication of Cultured Meat with Authentic Structural Properties. Food Hydrocoll..

[255] Kawecki N.S., Norris S.C.P., Xu Y., Wu Y., Davis A.R., Fridman E., Chen K.K., Crosbie R.H., Garmyn A.J., Li S. (2023). Engineering Multicomponent Tissue by Spontaneous Adhesion of Myogenic and Adipogenic Microtissues Cultured with Customized Scaffolds. Food Res. Int..

[256] Ong K.J., Johnston J., Datar I., Sewalt V., Holmes D., Shatkin J.A. (2021). Food Safety Considerations and Research Priorities for the Cultured Meat and Seafood Industry. Compr. Rev. Food Sci. Food Saf..

[257] Lee M., Choi W., Lee J.M., Lee S.T., Koh W.-G., Hong J. (2024). Flavor-Switchable Scaffold for Cultured Meat with Enhanced Aromatic Properties. Nat. Commun..

[258] Handral H.K., Tay S.H., Chan W.W., Choudhury D. (2022). 3D Printing of Cultured Meat Products. Crit. Rev. Food Sci. Nutr..

[259] Horgan G.W., Scalco A., Craig T., Whybrow S., Macdiarmid J.I. (2019). Social, Temporal and Situational Influences on Meat Consumption in the UK Population. Appetite.

[260] Quevedo-Silva F., Pereira J.B. (2022). Factors Affecting Consumers’ Cultivated Meat Purchase Intentions. Sustainability.

[261] Pakseresht A., Kaliji S.A., Canavari M. (2022). Review of Factors Affecting Consumer Acceptance of Cultured Meat. Appetite.

[262] Siegrist M., Hartmann C. (2020). Perceived Naturalness, Disgust, Trust and Food Neophobia as Predictors of Cultured Meat Acceptance in Ten Countries. Appetite.

[263] Bryant C., Sanctorum H. (2021). Alternative Proteins, Evolving Attitudes: Comparing Consumer Attitudes to Plant-Based and Cultured Meat in Belgium in Two Consecutive Years. Appetite.

[264] Klöckner C.A., Engel L., Moritz J., Burton R.J., Young J.F., Kidmose U., Ryynänen T. (2022). Milk, Meat, and Fish from the Petri Dish—Which Attributes Would Make Cultured Proteins (Un)Attractive and for Whom? Results From a Nordic Survey. Front. Sustain. Food Syst..

[265] Mancini M.C., Antonioli F. (2019). Exploring Consumers’ Attitude towards Cultured Meat in Italy. Meat Sci..

[266] Weinrich R., Strack M., Neugebauer F. (2020). Consumer Acceptance of Cultured Meat in Germany. Meat Sci..

[267] Dean D., Rombach M., Vriesekoop F., de Koning W., Aguiar L.K., Anderson M., Mongondry P., Urbano B., Luciano C.A.G., Jiang B. (2024). Should I Really Pay a Premium for This? Consumer Perspectives on Cultured Muscle, Plant-Based and Fungal-Based Protein as Meat Alternatives. J. Int. Food Agribus. Mark..

[268] Giezenaar C., Godfrey A.J.R., Ogilvie O.J., Coetzee P., Weerawarna N.R.P.M., Foster M., Hort J. (2023). Perceptions of Cultivated Meat in Millennial and Generation X Consumers Resident in Aotearoa New Zealand. Sustainability.

[269] Kerslake E., Kemper J.A., Conroy D. (2022). What’s Your Beef with Meat Substitutes? Exploring Barriers and Facilitators for Meat Substitutes in Omnivores, Vegetarians, and Vegans. Appetite.

[270] Vita G.D., Blanc S., Brun F., Bracco S., D’Amico M. (2019). Quality Attributes and Harmful Components of Cured Meats: Exploring the Attitudes of Italian Consumers towards Healthier Cooked Ham. Meat Sci..

[271] Melios S., Grasso S., Bolton D., Crofton E. (2024). Sensory Quality and Consumer Perception of Reduced/Free-from Nitrates/Nitrites Cured Meats. Curr. Opin. Food Sci..

[272] Brennan R., McGrath H., Canning L. (2024). Business-to-Business Marketing.

[273] Ahmad N.A., Arshad F., Nurul Azian Zakaria S., Ahmed M.U. (2023). A Review of Cultured Meat and Its Current Public Perception. Curr. Nutr. Food Sci..

[274] Bryant C., Dillard C. (2019). The Impact of Framing on Acceptance of Cultured Meat. Front. Nutr..

[275] de Koning W., Dean D., Vriesekoop F., Aguiar L.K., Anderson M., Mongondry P., Oppong-Gyamfi M., Urbano B., Luciano C.A.G., Jiang B. (2020). Drivers and Inhibitors in the Acceptance of Meat Alternatives: The Case of Plant and Insect-Based Proteins. Foods.

[276] Escribano A.J., Peña M.B., Díaz-Caro C., Elghannam A., Crespo-Cebada E., Mesías F.J. (2021). Stated Preferences for Plant-Based and Cultured Meat: A Choice Experiment Study of Spanish Consumers. Sustainability.

[277] Franceković P., García-Torralba L., Sakoulogeorga E., Vučković T., Perez-Cueto F.J.A. (2021). How Do Consumers Perceive Cultured Meat in Croatia, Greece, and Spain?. Nutrients.

[278] Gómez-Luciano C.A., de Aguiar L.K., Vriesekoop F., Urbano B. (2019). Consumers’ Willingness to Purchase Three Alternatives to Meat Proteins in the United Kingdom, Spain, Brazil and the Dominican Republic. Food Qual. Prefer..

[279] Bryant C., Szejda K., Parekh N., Deshpande V., Tse B. (2019). A Survey of Consumer Perceptions of Plant-Based and Clean Meat in the USA, India, and China. Front. Sustain. Food Syst..

[280] Engel L., Vilhelmsen K., Richter I., Moritz J., Ryynänen T., Young J.F., Burton R.J.F., Kidmose U., Klöckner C.A. (2024). Psychological Factors Influencing Consumer Intentions to Consume Cultured Meat, Fish and Dairy. Appetite.

[281] Hocquette É., Liu J., Ellies-Oury M.-P., Chriki S., Hocquette J.-F. (2022). Does the Future of Meat in France Depend on Cultured Muscle Cells? Answers from Different Consumer Segments. Meat Sci..

[282] Jacobs A.-K., Windhorst H.-W., Gickel J., Chriki S., Hocquette J.-F., Ellies-Oury M.-P. (2024). German Consumers’ Attitudes toward Artificial Meat. Front. Nutr..

[283] Kombolo Ngah M., Chriki S., Ellies-Oury M.-P., Liu J., Hocquette J.-F. (2023). Consumer Perception of “Artificial Meat” in the Educated Young and Urban Population of Africa. Front. Nutr..

[284] Ruzgys S., Pickering G.J. (2020). Perceptions of Cultured Meat Among Youth and Messaging Strategies. Front. Sustain. Food Syst..

[285] Bryant C., Barnett J. (2020). Consumer Acceptance of Cultured Meat: An Updated Review (2018–2020). Appl. Sci..

[286] Mendes G., Biscarra-Bellio J.C., Heidemann M.S., Taconeli C.A., Molento C.F.M. (2025). How Much Do Opinions Regarding Cultivated Meat Vary within the Same Country? The Cases of São Paulo and Salvador, Brazil. PLoS ONE.

[287] Ford H., Gould J., Danner L., Bastian S.E.P., Yang Q. (2023). “I Guess It’s Quite Trendy”: A Qualitative Insight into Young Meat-Eaters’ Sustainable Food Consumption Habits and Perceptions towards Current and Future Protein Alternatives. Appetite.

[288] Anant N., Pillay A., Juraimi S.A., Sheen F., Fogel A., Chong M.F.-F., Smith B.P.C., Pink A.E. (2025). “It’s Most Likely Gonna Be the Future”: A Qualitative Study Exploring Child and Parent Perceptions of Alternative Proteins. Appetite.

[289] de Valente J.P.S., Fiedler R.A., Sucha Heidemann M., Molento C.F.M. (2019). First Glimpse on Attitudes of Highly Educated Consumers towards Cell-Based Meat and Related Issues in Brazil. PLoS ONE.

[290] Szejda K., Bryant C.J., Urbanovich T. (2021). US and UK Consumer Adoption of Cultivated Meat: A Segmentation Study. Foods.

[291] Tomiyama A.J., Kawecki N.S., Rosenfeld D.L., Jay J.A., Rajagopal D., Rowat A.C. (2020). Bridging the Gap between the Science of Cultured Meat and Public Perceptions. Trends Food Sci. Technol..

[292] Rolland N.C.M., Markus C.R., Post M.J. (2020). The Effect of Information Content on Acceptance of Cultured Meat in a Tasting Context. PLoS ONE.

[293] Bryant C.J., Barnett J.C. (2019). What’s in a Name? Consumer Perceptions of in Vitro Meat under Different Names. Appetite.

[294] Nezlek J.B., Forestell C.A. (2019). Food Neophobia and the Five Factor Model of Personality. Food Qual. Prefer..

[295] Hwang J., You J., Moon J., Jeong J. (2020). Factors Affecting Consumers’ Alternative Meats Buying Intentions: Plant-Based Meat Alternative and Cultured Meat. Sustainability.

[296] Hartmann C., Furtwaengler P., Siegrist M. (2022). Consumers’ Evaluation of the Environmental Friendliness, Healthiness and Naturalness of Meat, Meat Substitutes, and Other Protein-Rich Foods. Food Qual. Prefer..

[297] Pasqualone A. (2022). Balancing Innovation and Neophobia in the Production of Food for Plant-Based Diets. Foods.

[298] Warner R.D. (2019). Review: Analysis of the Process and Drivers for Cellular Meat Production. Animal.

[299] Chia A., Shou Y., Wong N.M.Y., Cameron-Smith D., Sim X., Dam R.M.V., Chong M.F.-F. (2024). Complexity of Consumer Acceptance to Alternative Protein Foods in a Multiethnic Asian Population: A Comparison of Plant-Based Meat Alternatives, Cultured Meat, and Insect-Based Products. Food Qual. Prefer..

[300] Rombach M., Dean D., Vriesekoop F., de Koning W., Aguiar L.K., Anderson M., Mongondry P., Oppong-Gyamfi M., Urbano B., Luciano C.A.G. (2022). Is Cultured Meat a Promising Consumer Alternative? Exploring Key Factors Determining Consumer’s Willingness to Try, Buy and Pay a Premium for Cultured Meat. Appetite.

[301] Melios S., Grasso S. (2024). Meat Fans’ and Meat Reducers’ Attitudes towards Meat Consumption and Hybrid Meat Products in the UK: A Cluster Analysis. Int. J. Food Sci. Technol..

[302] Dagevos H. (2021). Finding Flexitarians: Current Studies on Meat Eaters and Meat Reducers. Trends Food Sci. Technol..

[303] Faccio E., Guiotto Nai Fovino L. (2019). Food Neophobia or Distrust of Novelties? Exploring Consumers’ Attitudes toward GMOs, Insects and Cultured Meat. Appl. Sci..

[304] Reis G.G., Heidemann M.S., Borini F.M., Molento C.F.M. (2020). Livestock Value Chain in Transition: Cultivated (Cell-Based) Meat and the Need for Breakthrough Capabilities. Technol. Soc..

[305] Bryant C.J. (2020). Culture, Meat, and Cultured Meat. J. Anim. Sci..

[306] Etter B., Michel F., Siegrist M. (2024). Which Are the Most Promising Protein Sources for Meat Alternatives?. Food Qual. Prefer..

[307] Yu Y., Wassmann B., Lanz M., Siegrist M. (2025). Willingness to Consume Cultured Meat: A Meta-Analysis. Trends Food Sci. Technol..

[308] Dagevos H., Verbeke W. (2022). Meat Consumption and Flexitarianism in the Low Countries. Meat Sci..

[309] Tonsor G.T., Lusk J.L. (2022). U.S. Perspective: Meat Demand Outdoes Meat Avoidance. Meat Sci..

[310] Font-i-Furnols M., Guerrero L. (2022). Spanish Perspective on Meat Consumption and Consumer Attitudes. Meat Sci..

[311] Ngapo T.M. (2022). Meat Analogues, the Canadian Meat Industry and the Canadian Consumer. Meat Sci..

[312] Realini C.E., Ares G., Antúnez L., Brito G., Luzardo S., del Campo M., Saunders C., Farouk M.M., Montossi F.M. (2022). Meat Insights: Uruguayan Consumers’ Mental Associations and Motives Underlying Consumption Changes. Meat Sci..

[313] Hötzel M.J., Vandresen B. (2022). Brazilians’ Attitudes to Meat Consumption and Production: Present and Future Challenges to the Sustainability of the Meat Industry. Meat Sci..

[314] Liu J., Hocquette É., Ellies-Oury M.-P., Chriki S., Hocquette J.-F. (2021). Chinese Consumers’ Attitudes and Potential Acceptance toward Artificial Meat. Foods.

[315] Dupont J., Fiebelkorn F. (2020). Attitudes and Acceptance of Young People toward the Consumption of Insects and Cultured Meat in Germany. Food Qual. Prefer..

[316] Choudhury D., Tseng T.W., Swartz E. (2020). The Business of Cultured Meat. Trends Biotechnol..

[317] Wilks M., Hornsey M., Bloom P. (2021). What Does It Mean to Say That Cultured Meat Is Unnatural?. Appetite.

[318] Rosenfeld D.L., Tomiyama A.J. (2022). Would You Eat a Burger Made in a Petri Dish? Why People Feel Disgusted by Cultured Meat. J. Environ. Psychol..

[319] Harris J., Ladak A., Mathur M.B. (2022). The Effects of Exposure to Information About Animal Welfare Reforms on Animal Farming Opposition: A Randomized Experiment. Anthrozoös.

[320] Kamalapuram S.K., Handral H., Choudhury D. (2021). Cultured Meat Prospects for a Billion!. Foods.

[321] Broom D.M. (2021). A Method for Assessing Sustainability, with Beef Production as an Example. Biol. Rev..

[322] Simões J., Moran D., Edwards S., Bonnet C., Lopez-Sebastian A., Chemineau P. (2021). Editorial: Sustainable Livestock Systems for High-Producing Animals. Animal.

[323] Abbate S., Centobelli P., Cerchione R. (2023). The Digital and Sustainable Transition of the Agri-Food Sector. Technol. Forecast. Soc. Change.

[324] Aschemann-Witzel J., Mulders M.D.G.H., Mouritzen S.L.T. (2023). Outside-in and Bottom-up: Using Sustainability Transitions to Understand the Development Phases of Mainstreaming Plant-Based in the Food Sector in a Meat and Dairy Focused Economy. Technol. Forecast. Soc. Change.

[325] Cassia F., Magno F. (2024). The Value of Self-Determination Theory in Marketing Studies: Insights from the Application of PLS-SEM and NCA to Anti-Food Waste Apps. J. Bus. Res..

[326] Mas F.D., Massaro M., Ndou V., Raguseo E. (2023). Blockchain Technologies for Sustainability in the Agrifood Sector: A Literature Review of Academic Research and Business Perspectives. Technol. Forecast. Soc. Change.

[327] Piancharoenwong A., Badir Y.F. (2024). IoT Smart Farming Adoption Intention under Climate Change: The Gain and Loss Perspective. Technol. Forecast. Soc. Change.

[328] Castellani P., Cassia F., Vargas-Sánchez A., Giaretta E. (2025). Food Innovation towards a Sustainable World: A Study on Intention to Purchase Lab-Grown Meat. Technol. Forecast. Soc. Change.

[329] Willett W., Rockström J., Loken B., Springmann M., Lang T., Vermeulen S., Garnett T., Tilman D., DeClerck F., Wood A. (2019). Food in the Anthropocene: The EAT–Lancet Commission on Healthy Diets from Sustainable Food Systems. Lancet.

[330] Croney C., Swanson J. (2023). Is Meat Eating Morally Defensible? Contemporary Ethical Considerations. Anim. Front..

[331] Aubin J., Vieux F., Féon S.L., Tharrey M., Peyraud J.L., Darmon N. (2025). Environmental Trade-Offs of Meeting Nutritional Requirements with a Lower Share of Animal Protein for Adult Subpopulations. Animal.

[332] González N., Marquès M., Nadal M., Domingo J.L. (2020). Meat Consumption: Which Are the Current Global Risks? A Review of Recent (2010–2020) Evidences. Food Res. Int..

[333] Xu X., Sharma P., Shu S., Lin T.-S., Ciais P., Tubiello F.N., Smith P., Campbell N., Jain A.K. (2021). Global Greenhouse Gas Emissions from Animal-Based Foods Are Twice Those of Plant-Based Foods. Nat. Food.

[334] Salzano A., D’Occhio M.J., Balestrieri A., Bifulco G., Limone A., Campanile G. (2025). Nutritional, Environmental and Social Profiles of Natural Meat and Food Derived from Cultured Muscle Cells: An Overview. Meat Sci..

[335] Huang W., Yin M., Xia J., Zhang X. (2024). A Review of Cross-Scale and Cross-Modal Intelligent Sensing and Detection Technology for Food Quality: Mechanism Analysis, Decoupling Strategy and Integrated Applications. Trends Food Sci. Technol..

[336] Madl L., Chang C., Innocenti M., Bargu S. (2025). Ethical Impact Assessment of Cultured Meat and Seafood.

[337] Helliwell R., Burton R.J.F. (2021). The Promised Land? Exploring the Future Visions and Narrative Silences of Cellular Agriculture in News and Industry Media. J. Rural Stud..

[338] Bakhsh A., Kim B., Ishamri I., Choi S., Li X., Li Q., Hur S.J., Park S. (2025). Cell-Based Meat Safety and Regulatory Approaches: A Comprehensive Review. Food Sci. Anim. Resour..

[339] Azhar A., Zeyaullah M., Bhunia S., Kacham S., Patil G., Muzammil K., Khan M.S., Sharma S. (2023). Cell-Based Meat: The Molecular Aspect. Front. Food Sci. Technol..

[340] Poirier N., Russell J. (2019). Does In Vitro Meat Constitute Animal Liberation?. J. Anim. Ethics.

[341] Kolkmann A.M., Van Essen A., Post M.J., Moutsatsou P. (2022). Development of a Chemically Defined Medium for in Vitro Expansion of Primary Bovine Satellite Cells. Front. Bioeng. Biotechnol..

[342] Kenigsberg J.A., Zivotofsky A.Z. (2020). A Jewish Religious Perspective on Cellular Agriculture. Front. Sustain. Food Syst..

[343] Lee A. (2018). Meat-Ing Demand: Is In Vitro Meat a Pragmatic, Problematic, or Paradoxical Solution?. Can. J. Women Law.

[344] Pajčin I., Knežić T., Savic Azoulay I., Vlajkov V., Djisalov M., Janjušević L., Grahovac J., Gadjanski I. (2022). Bioengineering Outlook on Cultivated Meat Production. Micromachines.

[345] Lim T., Chang H., Saad M.K., Joyce C.M., Park B., O’Beirne S.X., Cohen M.A., Kaplan D.L. (2024). Development of Serum-Reduced Medium for Mackerel Muscle Cell Line Cultivation. ACS Sustain. Chem. Eng..

[346] Mi J., Koh H.S.A., Srinivas V., Birch W.R., Zhou W. (2025). Towards Affordable Cultivated Meat: The Potential of Plant Protein Hydrolysates. Trends Food Sci. Technol..

[347] Bomkamp C., Carter M., Cohen M., Gertner D., Ignaszewski E., Murray S., O’Donnell M., Pierce B., Swartz E., Voss S. (2022). State of the Industry Report: Cultivated Meat and Seafood. Good Food Inst. GFI Retrieved June.

[348] Lee D.Y., Lee S.Y., Jung J.W., Kim J.H., Oh D.H., Kim H.W., Kang J.H., Choi J.S., Kim G.-D., Joo S.-T. (2023). Review of Technology and Materials for the Development of Cultured Meat. Crit. Rev. Food Sci. Nutr..

[349] Melios S., Gkatzionis K., Liu J., Ellies-Oury M.-P., Chriki S., Hocquette J.-F. (2025). Potential Cultured Meat Consumers in Greece: Attitudes, Motives, and Attributes Shaping Perceptions. Future Foods.

[350] Gross T. (2021). Novel Food Regulation in Israel—From Directive to Regulation.

[351] Formici G. (2023). Meating the Future: Alcune Riflessioni Sulla Necessità Di Promuovere Un Attento Dibattito Regolatorio in Materia Di Cd Carne Sintetica. Forum Quad. Cost..

[352] SFA (2024). Guidelines on Applying for Pre-Market Approval for a Novel Food.

[353] FDA (2022). FDA Completes First Pre-Market Consultation for Human Food Made Using Animal Cell Culture Technology.

[354] Health Canada (2022). Guidelines for the Safety Assessment of Novel Foods.

[355] Afonso A.L., Gelbmann W., Germini A., Fernández E.N., Parrino L., Precup G., Ververis E., European Food Safety Authority (2024). EFSA Scientific Colloquium 27: Cell Culture-derived Foods and Food Ingredients. EFSA Support. Publ..

[356] Senatto Della Repubblica (2023). Atto Senato Disposizioni in Materia di Divieto di Produzione e di Immissione Sul Mercato di Alimenti e Mangimi Sintetici.

[357] Israel—Ministry of Health Novel Food in Israel. https://www.gov.il/en/pages/novel-food.

[358] FAO (2024). Cell-Based Food and Precision Fermentation—Products, Safety and the Future Role. Stakeholder Roundtable Meeting Report.

[359] Gomez Romero S., Boyle N. (2023). Systems Biology and Metabolic Modeling for Cultivated Meat: A Promising Approach for Cell Culture Media Optimization and Cost Reduction. Compr. Rev. Food Sci. Food Saf..

[360] Maramraju S., Kowalczewski A., Kaza A., Liu X., Singaraju J.P., Albert M.V., Ma Z., Yang H. (2024). AI-Organoid Integrated Systems for Biomedical Studies and Applications. Bioeng. Transl. Med..

[361] Qiu L. (2023). U.S. Approves the Sale of Lab-Grown Chicken. The New York Times. Section B. Page 3. https://www.nytimes.com/2023/06/21/us/lab-grown-meat-sale-approval.html.

[362] Marquez A.S., Messer E., Gerber S., Cash S.B. (2025). “It’s Supposed to Be Real Meat”—An Analysis of Media Coverage of the First United States Sales Approval of Cell-Cultivated Chicken. Future Foods.

[363] Gorman A. (2025). Australia’s First Lab-Grown Meat Will Be on Menus within Weeks.

[364] Chiu A. (2025). No Bones, No Scales, No Problem: The First Lab-Grown Salmon Sold in the U.S. Wash. Post.

[365] Tsvakirai C.Z. (2024). The Valency of Consumers’ Perceptions toward Cultured Meat: A Review. Heliyon.

[366] Palmieri N., Perito M.A., Lupi C. (2020). Consumer Acceptance of Cultured Meat: Some Hints from Italy. Br. Food J..

[367] Wang O., Scrimgeour F. (2022). Consumer Segmentation and Motives for Choice of Cultured Meat in Two Chinese Cities: Shanghai and Chengdu. Br. Food J..

[368] Hamad A., Tayel A. (2025). Food 2050 Concept: Trends That Shape the Future of Food. J. Future Foods.

[369] Suntornnond R., Yap W.S., Lim P.Y., Choudhury D. (2024). Redefining the Plate: Biofabrication in Cultivated Meat for Sustainability, Cost Efficiency, Nutrient Enrichment, and Enhanced Organoleptic Experiences. Curr. Opin. Food Sci..

[370] Chriki S., Ellies-Oury M.-P., Hocquette J.-F. (2022). Is “Cultured Meat” a Viable Alternative to Slaughtering Animals and a Good Comprise between Animal Welfare and Human Expectations?. Anim. Front..

[371] Hocquette J.-F., Chriki S., Fournier D., Ellies-Oury M.-P. (2025). Review: Will “Cultured Meat” Transform Our Food System towards More Sustainability?. Animal.

[372] Tavan M., Smith N.W., McNabb W.C., Wood P. (2025). Reassessing the Sustainability Promise of Cultured Meat: A Critical Review with New Data Perspectives. Crit. Rev. Food Sci. Nutr..

[373] Wood P., Thorrez L., Hocquette J.-F., Troy D., Gagaoua M. (2023). “Cellular Agriculture”: Current Gaps between Facts and Claims Regarding “Cell-Based Meat”. Anim. Front..

[374] Loo E.J.V., Caputo V., Lusk J.L. (2020). Consumer Preferences for Farm-Raised Meat, Lab-Grown Meat, and Plant-Based Meat Alternatives: Does Information or Brand Matter?. Food Policy.

[375] Stephens N. (2022). Join Our Team, Change the World: Edibility, Producibility and Food Futures in Cultured Meat Company Recruitment Videos. Food Cult. Soc..

[376] Newman L., Katz-Rosene R.M., Martin S.J. (2020). The promise and peril of “cultured meat”. In Green Meat?: Sustaining Eaters Animals and the Planet.

[377] Ye Y., Zhou J., Guan X., Sun X. (2022). Commercialization of Cultured Meat Products: Current Status, Challenges, and Strategic Prospects. Future Foods.

[378] Bushnell C., Specht L., Almy J. (2022). Cultivated Meat and Seafood. State of the Industry Report.

[379] Tsvakirai C.Z., Nalley L.L., Tshehla M. (2024). What Do We Know about Consumers’ Attitudes towards Cultured Meat? A Scoping Review. Future Foods.

[380] Tuomisto H.L. (2022). Challenges of Assessing the Environmental Sustainability of Cellular Agriculture. Nat. Food.

[381] Smetana S., Ristic D., Pleissner D., Tuomisto H.L., Parniakov O., Heinz V. (2023). Meat Substitutes: Resource Demands and Environmental Footprints. Resour. Conserv. Recycl..

[382] Kirsch M., Morales-Dalmau J., Lavrentieva A. (2023). Cultivated Meat Manufacturing: Technology, Trends, and Challenges. Eng. Life Sci..

[383] Flaws B. (2019). Fonterra Is Investing in Artificial Meat, But Would You Eat It.

[384] Terazono E. (2021). Lab-Grown Chicken Start-up Slashes Production Costs. Financial Times. 452-f3e0. https://www.ft.com/content/ae4dd452-f3e0-4a38-a29d-3516c5280bc7.

[385] Garrison G.L., Biermacher J.T., Brorsen B.W. (2022). How Much Will Large-Scale Production of Cell-Cultured Meat Cost?. J. Agric. Food Res..

[386] Pasitka L., Wissotsky G., Ayyash M., Yarza N., Rosoff G., Kaminker R., Nahmias Y. (2024). Empirical Economic Analysis Shows Cost-Effective Continuous Manufacturing of Cultivated Chicken Using Animal-Free Medium. Nat. Food.

[387] Bambridge-Sutton A. Gourmey Cuts Cultivated Meat Costs to €7/Kg 2025.

[388] Mridul A. Could AI Be the Solution for Cheap Cultivated Meat? This French Startup Is Betting on It 2025.

[389] Bell D. Supermarkets and Cultivated Meat: What to Know 2026.

[390] Djisalov M., Knežić T., Podunavac I., Živojević K., Radonic V., Knežević N.Ž., Bobrinetskiy I., Gadjanski I. (2021). Cultivating Multidisciplinarity: Manufacturing and Sensing Challenges in Cultured Meat Production. Biology.

[391] Patil R.A., Bhavana A., Patil B.R. (2024). Cultured Meat: The Upcoming Meat Production Having Sustainable Benefits over Conventional Meat Production: A Review. Agric. Rev..

[392] Hossain M.A., Ellahi R.M., Alam F. (2025). Dissecting the Cultured Meat Supply Chain: A Comprehensive Review. Trends Food Sci. Technol..

[393] Bhat Z.F., Morton J.D., Bekhit A.E.-D.A., Kumar S., Bhat H.F. (2022). Cultured Meat: Challenges in the Path of Production and 3D Food Printing as an Option to Develop Cultured Meat-Based Products. Alternative Proteins.

[394] Nobre F.S. (2022). Cultured Meat and the Sustainable Development Goals. Trends Food Sci. Technol..

[395] US FDA (2020). Freedom of Information Summary—Original New Animal Drug Application: NADA 141–542 pPL657 rDNA Construct in Domestic Pigs.

[396] Furuhashi M., Morimoto Y., Shima A., Nakamura F., Ishikawa H., Takeuchi S. (2021). Formation of Contractile 3D Bovine Muscle Tissue for Construction of Millimetre-Thick Cultured Steak. npj Sci. Food.

[397] Gasteratos K. (2019). 90 Reasons to Consider Cellular Agriculture.

[398] Olenic M., Thorrez L. (2023). Cultured Meat Production: What We Know, What We Don’t Know and What We Should Know. Ital. J. Anim. Sci..

[399] Ramezani P., Motamedzadegan A. (2024). Exploring the Potential of Cultured Meat: Technological Advancements, Sustainability Prospects, and Challenges. Iran. Food Sci. Technol. Res. J..

[400] Stout A.J. (2023). Strategies for Enhanced Cultured Meat Products and Processes.

[401] Bui H.T., Filimonau V., Ermolaev V.A. (2025). Cultivated Meat in Tourism and Hospitality: Setting the Scene and Outlining Future Research Agenda. J. Foodserv. Bus. Res..

[402] Morais-da-Silva R.L., Reis G.G., Sanctorum H., Molento C.F.M. (2022). The Social Impacts of a Transition from Conventional to Cultivated and Plant-Based Meats: Evidence from Brazil. Food Policy.

[403] McClements D.J. (2023). Ultraprocessed Plant-Based Foods: Designing the next Generation of Healthy and Sustainable Alternatives to Animal-Based Foods. Compr. Rev. Food Sci. Food Saf..

[404] Kaliji S.A., Pakseresht A., Hocquette J.-F. (2025). Can Blockchain Revolutionize Meat Production? Addressing Transparency, Trust, and Compliance in Conventional and Cultured Meat. Trends Food Sci. Technol..

[405] Brooks S. (2021). Configuring the Digital Farmer: A Nudge World in the Making?. Econ. Soc..

[406] Duncan E., Rotz S., Magnan A., Bronson K. (2022). Disciplining Land through Data: The Role of Agricultural Technologies in Farmland Assetisation. Sociol. Rural..

[407] Siegrist M., Michel F., Hartmann C. (2024). The Shift from Meat to Plant-Based Proteins: Consumers and Public Policy. Curr. Opin. Food Sci..

[408] Barone P.W., Wiebe M.E., Leung J.C., Hussein I.T.M., Keumurian F.J., Bouressa J., Brussel A., Chen D., Chong M., Dehghani H. (2020). Viral Contamination in Biologic Manufacture and Implications for Emerging Therapies. Nat. Biotechnol..

[409] Reddy B.L., Saier M.H. (2020). The Causal Relationship between Eating Animals and Viral Epidemics. Microb. Physiol..

[410] Welch D., Swartz E. (2020). Food Safety Considerations for Cultivated Meat.

[411] Habowski K., Sant’Ana A.S. (2024). Microbiology of Cultivated Meat: What Do We Know and What We Still Need to Know?. Trends Food Sci. Technol..

[412] Jaime-Rodríguez M., Cadena-Hernández A.L., Rosales-Valencia L.D., Padilla-Sánchez J.M., Chavez-Santoscoy R.A. (2023). Are Genetic Drift and Stem Cell Adherence in Laboratory Culture Issues for Cultivated Meat Production?. Front. Nutr..

[413] Yang D.H., Kook K.-S., Heo Y., Kim W.-J. (2025). Future Protein Alternative: Recent Progress and Challenges in Cellular Agriculture. Food Sci. Biotechnol..

[414] Manning L. (2024). Responsible Innovation: Mitigating the Food Safety Aspects of Cultured Meat Production. J. Food Sci..

[415] Guo Y., Ding S.-J., Ding X., Liu Z., Wang J.-L., Chen Y., Liu P.-P., Li H.-X., Zhou G.-H., Tang C.-B. (2022). Effects of Selected Flavonoids Oncellproliferation and Differentiation of Porcine Muscle Stem Cells for Cultured Meat Production. Food Res. Int..

[416] Ong S., Loo L., Pang M., Tan R., Teng Y., Lou X., Chin S.K., Naik M.Y., Yu H. (2021). Decompartmentalisation as a Simple Color Manipulation of Plant-Based Marbling Meat Alternatives. Biomaterials.

[417] Yun S.H., Lee D.Y., Lee S.Y., Lee J., Mariano E.J., Joo S.-T., Choi I., Choi J.S., Kim G.-D., Hur S.J. (2023). Improved Culture Procedure for Bovine Muscle Satellite Cells for Cultured Meat. Food Res. Int..

[418] Banach J.L., van der Berg J.P., Kleter G., van Bokhorst-van de Veen H., Bastiaan-Net S., Pouvreau L., van Asselt E.D. (2023). Alternative Proteins for Meat and Dairy Replacers: Food Safety and Future Trends. Crit. Rev. Food Sci. Nutr..

[419] Turck D., Bohn T., Castenmiller J., de Henauw S., Hirsch-Ernst K.I., Maciuk A., Mangelsdorf I., McArdle H.J., Naska A., EFSA Panel on Nutrition, Novel Foods and Food Allergens (NDA) (2024). Guidance on the Scientific Requirements for an Application for Authorisation of a Novel Food in the Context of Regulation (EU) 2015/2283. EFSA J..

[420] Lee L.Y.G.N., Leow S.Y., Wen H., Soh J.Y., Chiang W.C., Zhong Y., Tham E.H., Loh W., Delsing D.J., Lee B.W. (2022). An Evaluation of the Mechanisms of Galacto-Oligosaccharide (GOS)-Induced IgE Cross-Linking on Basophils in GOS Allergy. Front. Allergy.

[421] Commins S.P. (2020). Diagnosis & Management of Alpha-Gal Syndrome: Lessons from 2500 Patients. Expert Rev. Clin. Immunol..

[422] Anania C., Cuomo B., D’Auria E., Decimo F., Giannì G., Indirli G.C., Manca E., Mondì F., Pendezza E., Sartorio M.U.A. (2025). Sustainable Nutrition and Food Allergy: A State-of-the-Art Review. Nutrients.

[423] Tan Y.Q., Ong H.C., Yong A.M.H., Fattori V., Mukherjee K. (2024). Addressing the Safety of New Food Sources and Production Systems. Compr. Rev. Food Sci. Food Saf..

[424] Ong K.J., Case F., Shatkin J.A. (2024). Food Safety of Fermented Proteins and Cultivated Meat and Seafood. Cellular Agriculture.

[425] Li L., Chen L., Chen X., Chen Y., Ding S., Fan X., Liu Y., Xu X., Zhou G., Zhu B. (2022). Chitosan-sodium Alginate-Collagen/Gelatin Three-Dimensional Edible Scaffolds for Building a Structured Model for Cell Cultured Meat. Int. J. Biol. Macromol..

[426] Ng S., Kurisawa M. (2021). Integrating Biomaterials and Food Biopolymers for Cultured Meat Production. Acta Biomater..

[427] Sealy M.P., Avegnon K.L.M., Garrett A., Delbreilh L., Bapat S., Malshe A.P. (2022). Understanding Biomanufacturing of Soy-Based Scaffolds for Cell-Cultured Meat by Vat Polymerization. CIRP Ann..

[428] Hubalek S., Post M.J., Moutsatsou P. (2022). Towards Resource-Efficient and Cost-Efficient Cultured Meat. Curr. Opin. Food Sci..

[429] Zhang J., Liu L., Liu H., Yoon A., Rizvi S.S.H., Wang Q. (2019). Changes in Conformation and Quality of Vegetable Protein during Texturization Process by Extrusion. Crit. Rev. Food Sci. Nutr..

[430] Lee S.-H., Choi J. (2024). Three-Dimensional Scaffolds, Materials, and Fabrication for Cultured Meat Applications: A Scoping Review and Future Direction. Food Hydrocoll..

[431] Zhang K., Zang M., Wang S., Zhang Z., Li D., Li X. (2023). Development of Meat Analogs: Focus on the Current Status and Challenges of Regulatory Legislation. Compr. Rev. Food Sci. Food Saf..

[432] Engels W., Siu J., van Schalkwijk S., Wesselink W., Jacobs S., Bachmann H. (2022). Metabolic Conversions by Lactic Acid Bacteria during Plant Protein Fermentations. Foods.

[433] Kyrylenko A., Eijlander R.T., Alliney G., de Bos E.L., Wells-Bennik M.H.J. (2023). Levels and Types of Microbial Contaminants in Different Plant-Based Ingredients Used in Dairy Alternatives. Int. J. Food Microbiol..

[434] Sogore T., Guo M., Sun N., Jiang D., Shen M., Ding T. (2024). Microbiological and Chemical Hazards in Cultured Meat and Methods for Their Detection. Compr. Rev. Food Sci. Food Saf..

[435] Garcia E.E.C., Nabeshima E.H., Sadahira M.S., Ferrari R.A., da Silva N., Sarantopoulos C.I.G., Berbari S.A., Pacheco M.T.B. (2022). Estudo Regulatório Sobre Proteínas Alternativas No Brasil: Proteínas Vegetais.

[436] Failla M., Hopfer H., Wee J. (2023). Evaluation of Public Submissions to the USDA for Labeling of Cell-Cultured Meat in the United States. Front. Nutr..

[437] Stevens H., Ruperti Y. (2024). Smart Food: Novel Foods, Food Security, and the Smart Nation in Singapore. Food Cult. Soc..

